# Effect of TraN key residues involved in DNA binding on pIP501 transfer rates in *Enterococcus faecalis*

**DOI:** 10.3389/fmolb.2024.1268647

**Published:** 2024-02-06

**Authors:** Claudia Michaelis, Tamara M. I. Berger, Kirill Kuhlmann, Rangina Ghulam, Lukas Petrowitsch, Maria Besora Vecino, Bernd Gesslbauer, Tea Pavkov-Keller, Walter Keller, Elisabeth Grohmann

**Affiliations:** ^1^ Faculty of Life Sciences and Technology, Department of Microbiology, Berliner Hochschule für Technik, Berlin, Germany; ^2^ Institute of Molecular Biosciences, University of Graz, Graz, Austria; ^3^ Institute of Pharmaceutical Sciences, Division of Pharmaceutical Chemistry, University of Graz, Graz, Austria; ^4^ BioTechMed Graz, Graz, Austria; ^5^ Field of Excellence BioHealth—University of Graz, Graz, Austria

**Keywords:** type IV secretion system, antibiotic resistance, conjugative transfer, pIP501, *Enterococcus faecalis*, transfer regulator, DNA binding proteins

## Abstract

Conjugation is a major mechanism that facilitates the exchange of antibiotic resistance genes among bacteria. The broad-host-range Inc18 plasmid pIP501 harbors 15 genes that encode for a type IV secretion system (T4SS). It is a membrane-spanning multiprotein complex formed between conjugating donor and recipient cells. The penultimate gene of the pIP501 operon encodes for the cytosolic monomeric protein TraN. This acts as a transcriptional regulator by binding upstream of the operon promotor, partially overlapping with the origin of transfer. Additionally, TraN regulates *traN* and *traO* expression by binding upstream of the P_*traNO*_ promoter. This study investigates the impact of nine TraN amino acids involved in binding to pIP501 DNA through site-directed mutagenesis by exchanging one to three residues by alanine. For three *traN* variants, complementation of the pIP501∆*traN* knockout resulted in an increase of the transfer rate by more than 1.5 orders of magnitude compared to complementation of the mutant with native *traN.* Microscale thermophoresis (MST) was used to assess the binding affinities of three TraN double-substituted variants and one triple-substituted variant to its cognate pIP501 double-stranded DNA. The MST data strongly correlated with the transfer rates obtained by biparental mating assays in *Enterococcus faecalis*. The TraN variants TraN_R23A-N24A-Q28A, TraN_H82A-R86A, and TraN_G100A-K101A not only exhibited significantly lower DNA binding affinities but also, upon complementation of the pIP501∆*traN* knockout, resulted in the highest pIP501 transfer rates. This confirms the important role of the TraN residues R23, N24, Q28, H82, R86, G100, and K101 in downregulating pIP501 transfer. Although TraN is not part of the mating pair formation complex, TraE, TraF, TraH, TraJ, TraK, and TraM were coeluted with TraN in a pull-down. Moreover, TraN homologs are present not only in Inc18 plasmids but also in RepA_N and Rep_3 family plasmids, which are frequently found in enterococci, streptococci, and staphylococci. This points to a widespread role of this repressor in conjugative plasmid transfer among Firmicutes.

## 1 Introduction

The fundamental process of horizontal gene transfer (HGT) has greatly enhanced our understanding of microbial adaptation and the spread of antibiotic resistance genes (ARGs) as well as virulence factors among bacteria. The acquisition of new traits (drug resistance and metabolic and pathogenic capabilities) can confer selective advantages to bacteria and shape the evolution of microbial populations.

Conjugation is one of the major HGT mechanisms that enables the rapid transfer of ARGs from one bacterium to another. It is mediated by a membrane-embedded type IV secretion system (T4SS) that translocates single-stranded DNA from donor to recipient cells. Conjugation is a key mechanism in the development of multi-drug-resistant bacteria because it allows them to adapt to changing environments more efficiently (Li et al., 2019; Virolle et al., 2020; Costa et al., 2021). Conjugative T4SSs work in a contact-dependent manner: Gram-negative bacteria develop a pilus to initiate cell-to-cell contact between donor and recipient cells, while Gram-positive conjugation is thought to rely on adhesins on the donor cell surface. T4SSs have been studied in numerous Gram-negative bacterial species, but the understanding of Gram-positive T4SSs remains relatively limited (Grohmann et al., 2018; Costa et al., 2021). Mobile genetic elements (MGEs) such as conjugative plasmids and integrative and conjugative elements (ICEs) encode their own T4SS. One model plasmid used to study conjugative transfer in Gram-positive bacteria is the conjugative plasmid pIP501. This ∼30.6-kb plasmid belongs to incompatibility (Inc) group 18, whose representatives encode several resistance genes, such as linezolid or vancomycin resistance genes, or to the macrolide-lincosamide–streptogramine group of resistance genes (Kohler et al., 2018b; Cinthi et al., 2022; Tang et al., 2023). The plasmid can stably replicate and transfer into a variety of Gram-positive bacteria, including enterococci, streptococci, staphylococci, lactococci, *Oenococcus oeni*, pediococci, *Listeria*, *Leuconostoc mesenteroides* subsp*. cremoris*, lactobacilli, *Bacillus subtilis*, *Streptomyces lividan*s, and Gram-negative *Escherichia coli* (Pérez-Díaz et al., 1982; Schaberg et al., 1982; Gonzalez and Kunka, 1983; Buu-Hoï et al., 1984; Pucci et al., 1988; Thompson and Collins, 1988; Langella and Chopin, 1989; Kurenbach et al., 2003; Zúñiga et al., 2003). Enterococci, particularly *Enterococcus faecalis* and *E. faecium*, are nosocomial pathogens of great clinical importance. They can cause severe healthcare-associated infections such as urinary tract infections, bloodstream infections, or endocarditis (Haliga et al., 2023; Mancuso et al., 2023). Owing to their high acquisition of ARGs and consequent resistance to a broad spectrum of antibiotics, these infections are challenging to treat (Khan et al., 2022; Murray et al., 2022).

The conjugative T4SS of the broad-host-range plasmid pIP501 is encoded on a ∼15-kb transfer (*tra*) operon and comprises 15 transfer genes (*traA*–*traO*). Transfer proteins are involved in DNA processing, relaxosome recruitment, mating pore formation (MPF), and the regulation of the expression of the *tra* operon. The last gene of the operon encodes for TraO, a putative surface adhesin, which enhances the cell-to-cell contact between donor and recipient cells prior to conjugative transfer of the plasmid. Transcriptional regulation is critical for bacteria to minimize the energy and metabolic costs associated with generating the multiprotein complex required for pIP501 transfer. Regulation of the transcription of conjugative transfer genes leads to tighter control of the conjugative process. The transcriptional regulation of Gram-positive T4SSs has been observed for many conjugative plasmids and other MGEs and has recently been reviewed (Kohler et al., 2019). The transcriptional regulation of transfer is primarily mediated by plasmid-encoded regulatory proteins that specifically bind to or in the vicinity of the promoter region, leading to repression of the transcription of the transfer genes. Quorum-sensing mechanisms for expression control of transfer genes have been identified, such as for the conjugative elements pLS20 or ICE*Bs*1 from *B. subtilis* (Auchtung et al., 2005; Singh et al., 2013). In case of plasmid pCF10, the DNA binding activity of the repressor is further modulated by the ratio of two peptide sex pheromones, plasmid-encoded cCF10, and recipient pheromone iCF10 (Breuer et al., 2018).

The *tra* gene expression of pIP501 is regulated by the relaxase TraA, which binds to the main T4SS promoter, P_*tra*_ (Grohmann et al., 2016). The repressor TraN also acts as a transcriptional regulator by specifically binding to a preferred 34-bp sequence (original binding site [oBS]) located 69 bp upstream of the *oriT*_pIP501_; this serves as the origin of transfer where conjugative transfer is initiated. Furthermore, TraN binds to a second promoter, P_*traNO*_, to regulate its own but also TraO expression (Kohler et al., 2018a).

The crystal structure of TraN has already been solved (Goessweiner-Mohr et al., 2012; Goessweiner-Mohr et al., 2014a). Co-crystallization of TraN was conducted to examine the interactions between TraN and its cognate pIP501 binding site (Kohler et al., 2018a). Both crystal structures show TraN in its monomeric form. Sequence-specific interactions between distinct amino acid side chains and bases of pIP501 DNA were identified as important recognition sites for the strong and sequence-specific binding of TraN. Analogous TraN-like functions have been discovered in other Inc18 and related conjugative plasmids (Kohler et al., 2018a). Furthermore, it was demonstrated that TraN exhibits structural similarities to transcriptional regulators of the MerR family as well as to excisionases from bacteriophages and the conjugative element Tn*916* (Goessweiner-Mohr et al., 2014a).

This study aims to demonstrate the pivotal role of TraN key residues in sequence-specific DNA binding.

To determine the role of key amino acid residues of TraN in the regulation of pIP501 transfer, three single and four multiple alanine substitution variants were generated to complement the *traN* knockout in pIP501. The four TraN variants with the highest impact on pIP501 transfer were expressed in *E. coli* and purified. Microscale thermophoresis (MST) studies were performed to reveal the effect of TraN substitutions on DNA binding affinity. Furthermore, pull-down assays elucidated the potential T4SS interaction partners of TraN using immunoblotting and mass spectrometry (MS) to identify the coeluted Tra proteins.

## 2 Materials and methods

### 2.1 Bacterial strains and culture conditions

A list of all strains and plasmids is provided in Supplementary Table S1. *E. faecalis* strains were routinely grown on BHI plates or in BHI medium with shaking at 37°C. *E. coli* was grown at 37°C in LB medium with shaking. Antibiotics were used at the concentrations given in Supplementary Table S1.

### 2.2 Complementation of *traN* deletion in *E. faecalis* JH2-2 (pIP501∆*traN*)

The pIP501∆*traN* in-frame knockout constructed by Kohler et al. (2018a) was complemented with the wild-type *traN* containing its native ribosomal binding site (RBS). *TraN* with its native RBS was thus amplified from pIP501 using the primers pEU327-BstYI_RBS traN fw and pEU327_SalI RBS traN rev. The PCR fragment was digested with SalI and BstYI and cloned into the shuttle vector pEU327. All primers used for cloning are listed in Supplementary Table S2.

Seven pEU327-RBS-*traN* variants were constructed for further complementation studies. They were designed using site-directed mutagenesis (New England Biolabs, Frankfurt am Main, Germany) according to the manufacturer’s instructions and transformed into competent *E. coli* DH5α cells. The sequence of the *traN* variants was verified by Sanger sequencing. For biparental mating assays, *E. faecalis* JH2-2 (pIP501∆*traN*) was transformed by electroporation with one of the seven pEU327-RBS-*traN* variants as described in Berger et al. (2022).

### 2.3 Biparental mating assays

The biparental mating assays were performed as described previously (Berger et al., 2022). They were carried out in duplicates and repeated three times. pIP501 transfer rates are given as transconjugants per recipient cell. GraphPad Prism 8 statistical software was used for statistical analyses. Statistical significance was determined by using the unpaired two-tailed Mann–Whitney U test. Independent biological triplicates of knockout complementation by *traN* variants were compared with complementation by the native *traN* gene. *p-*values: *p* < 0.1 (*), *p* < 0.01 (**).

### 2.4 Cloning of *traN* variants into a 7×His-tag expression vector

To add an N-terminal 7×His-tag in-frame to the *traN* sequence, the *traN* variants were subcloned into the 7×His-tag expression vector pQTEV. For improved readability, we refer to them without including the “His” prefix throughout the article. Thus, a Gibson Assembly Kit (New England Biolabs) was applied according to the manufacturer’s instructions. The primers for amplification of the pQTEV backbone (GA_pQTEV189-190 fw and GA_pQTEV189-190 rev) and *traN* (GA_pQTEV-traN fw and GA_pQTEV-traN rev) as well as those for sequencing are listed in Supplementary Table S2. The recombinant plasmids were transformed into *E. coli* DH5α (New England Biolabs). The sequence of the *traN* variants was confirmed by Sanger sequencing (Eurofins Genomics Germany). For expression of TraN and TraN variants (TraN_R23A-N24A-Q28A, TraN_G47A-G47A, TraN_H82A-R86A, and TraN_G100A-K101A), the recombinant plasmids were transformed into *E. coli* Lemo21 (DE3) cells (New England Biolabs).

### 2.5 Expression levels of TraN variants in *E. faecalis* JH2-2

To compare expression levels of the seven TraN variants with that of TraN, *E. faecalis* JH2-2 (pIP501∆*traN*) strains harboring pEU327-RBS-*traN* variants and pEU327-RBS-*traN*, respectively, were cultured in 4 mL BHI medium supplemented with respective antibiotics overnight under constant shaking at 37°C. *E. faecalis* JH2-2 (pEU327) and *E. faecalis* JH2-2 (pIP501∆*traN*) served as negative controls for verifying the antibody specificity. The overnight cultures were normalized to an optical density of 600 nm (OD_600_) of 2 in a volume of 1 mL and harvested by centrifugation at 8,000 ×g for 15 min at 4°C. The cell pellets were washed three times in 1 mL phosphate buffer (50 mM KH_2_PO_4_/K_2_HPO_4_, pH 7) and resuspended in 300 µL SDS-PAGE loading buffer as per standard protocol (Laemmli, 1970). They were lysed by sonication through two pulses of 8 s followed by boiling at 95°C for 10 min. Identical amounts of the protein lysates were loaded onto a 12% SDS polyacrylamide gel. Western blots were probed with polyclonal anti-TraN antibody (BioGenes GmbH, Berlin, Germany) at 1:10,000 dilution followed by secondary horseradish peroxidase conjugated IgG antibody (Promega GmbH, Germany) at a dilution of 1:10,000. To check whether the TraN mutations impaired the affinity of the TraN antibody, we performed a Western blot with the heterologously expressed purified variants TraN_R23A-N24A-Q28A, TraN_G47A-G48A, TraN_H82A-R86A, and TraN_G100A-K101A, and the heterologously expressed TraN. We loaded 0.75 µg of each protein onto a 12% SDS polyacrylamide gel followed by immunoblotting, as described above.

### 2.6 Expression and affinity purification of His-tagged TraN and TraN variants

The same expression and purification protocol was applied to TraN and its variants. A multiple sequence alignment of all variants is shown in Supplementary Figure S1A.

We prepared 50 mL overnight cultures (ONC) supplemented with 0.1 mg/mL ampicillin either from a single colony of an agar plate or from a frozen cryostock. Three-liter LB broth supplemented with 0.1 mg/mL ampicillin was inoculated with the ONC to an initial OD_600_ of 0.05 and incubated under shaking at 37°C. Proteins were expressed after induction of the culture with 1 mM IPTG (final concentration) at an OD_600_ of 0.4–0.6. Incubation continued for 3 h at 37°C under shaking. The cells were harvested by centrifugation at 9,000 ×g (JLA 8.100, Beckman Coulter Inc., Brea, United States) and stored at −20°C.

The frozen cell pellets were resuspended in a triple amount of 50 mM HEPES pH 7.5, 100 mM (NH_4_)_2_SO_4_ supplemented with protease inhibitors (0.001 mg/mL pepstatin, 0.002 mg/mL antipain, and 0.02 mg/mL leupeptin final concentration; Merck KGaA, Darmstadt, Germany) in relation to the total net cell weight. The cell suspension was homogenized (T18 digital ULTRA-TURRAX^®^, IKA®-Werke GmbH & Co. KG, Staufen, Germany) and sonicated in a rosette cell on ice (T13 flat tip, 50% duty cycle, ∼60% amplitude, Sonopuls, UW/HD 2070, Bandelin electronic GmbH & Co. KG, Germany) twice for 10 min each. The cell debris were removed by centrifugation for 45 min at 4°C and 35,000 ×g.

The supernatants were filtered through 0.45 µm filters (Rotilabo® syringe filters, Carl-Roth GmbH + Co. KG, Karlsruhe, Germany) and loaded onto a HisTrap HP 5 mL column (Cytiva, Chalfont St. Giles, England) for affinity purification using the ÄKTA Pure system (Cytiva). The proteins were eluted with a 0.3 M imidazole step and a 0.3–1.5 M linear imidazole gradient. The quality of the samples was assessed by 12% SDS-PAGE, and the fractions containing most of the desired protein were pooled and loaded onto a 5-mL HeparinTrap column (Cytiva) to remove bound DNA. We applied 50 mM HEPES, 100 mM (NH_4_)_2_SO_4_ pH 7.5 as running buffer. The proteins were eluted with a NaCl gradient from 0 to 1.5 M. The eluting peak fractions containing most of the desired protein were pooled and concentrated using filter-insert spin tubes (Amicon^®^ Ultra centrifugal filters, Ultracell^®^ 3000 MWCO, Merck Millipore Ltd., Darmstadt, Germany).

Additional analytical gel filtration was performed with the ÄKTA avant system (Cytiva) to determine the purity and oligomerization state of TraN and its variants. For this, 500 μL protein samples were loaded onto a Sephadex 75 10/300 GL increase column (Cytiva) using a 2-mL loop and 50 mM HEPES, 100 mM (NH_4_)_2_SO_4_ pH 7.6 as running buffer. To check for DNA bound to the proteins, a chromatogram with two wavelengths was recorded at 280 nm for protein and 260 nm for DNA detection. Expression and purification were monitored by SDS-PAGE using 12% SDS polyacrylamide gels. To normalize the amount of loaded cell mass, we took samples equal to an OD_600_ of 2 in 1 mL and resuspended them in 20 µL SDS loading buffer.

### 2.7 Circular dichroism (CD) measurements

Purified TraN and its variants were changed into a phosphate buffer (50 mM NaH_2_PO_4_ pH 7.0, 50 mM NaF) by filter-insert spin tubes (Amicon^®^ Ultra centrifugal filters, Ultracell^®^ 3000 MWCO, Merck Millipore Ltd.). Protein concentrations between 0.08 mg/mL and 0.13 mg/mL were used. CD measurements were performed on a J-1500 circular dichroism spectrophotometer (JASCO Corporation, Tokyo, Japan). Far-UV CD spectra were recorded at 20°C from 260 to 190 nm with a data pitch of 0.2 nm and a bandwidth of 2 nm applying a scan speed of 50 nm/min. Ten spectra were recorded and accumulated for each sample. The resulting spectra were baseline-corrected by subtracting the signal of the buffer. The CD signal was converted to mean residue ellipticity [θ]_MRE_, and the secondary structure was determined with DichroWeb using the CDSSTR algorithm (Compton and Johnson, 1986; Whitmore and Wallace, 2004; 2008; Miles et al., 2022) and SMP180t reference dataset (Abdul-Gader et al., 2011). A weighted-means smoothing algorithm over 25 datapoints was applied to all spectra.

### 2.8 Generation of double-stranded oligonucleotides for MST

Two pIP501-specific double-stranded DNA (dsDNA) oligonucleotides were created by annealing their complementary single strands ordered from Thermo Fisher Scientific. The melting temperature (T_m_) of the oligonucleotides was calculated with SnapGene Viewer (SnapGene software, www.snapgene.com). The oligonucleotides corresponded to 1) a specific 34-mer (original TraN binding site (oBS), T_m_ = 56°C), and 2) a random 34-mer (*oriT*_pIP501_ region, to which no specific binding had been demonstrated (random DNA), T_m_ = 66°C) (Goessweiner-Mohr et al., 2014a).

The oligonucleotide sequences are given in Supplementary Table S2. The forward and reverse strands of the oligonucleotides were annealed before using them in MST. To this end, 30 µL of a 1 mM solution of each ss-oligonucleotide was combined. The mixture was kept at 95°C for 1 h in a heating block (Drybath Stdrd 1blck, Thermo Fisher Scientific) and then slowly cooled down in an enclosed Styrofoam box yielding 60 µL of 500 μM ds-oligonucleotide each.

### 2.9 Microscale thermophoresis (MST)

Monolith NT.115 (NanoTemper Technologies GmbH, Munich, Germany) was used to measure the protein-DNA interactions between TraN and TraN variants with the oBS and the random DNA. The generation of the ds-oligonucleotides is described above in 2.8.

Purified TraN and its variants were changed into phosphate-buffered saline with tween (PBS-T) buffer (137 mM NaCl, 2.7 mM KCl, 10 mM Na_2_HPO_4_, 1.8 mM KH_2_PO_4_, 0.05% Tween 20 (v/v)) by filter-insert spin tubes (Amicon^®^ Ultra Centrifugal Filters, Ultracell^®^ 3000 MWCO, Merck Millipore Ltd.). Protein concentrations were determined using NanoDrop (ND1000, PEQLAB Biotechnologie GmbH, Erlangen, Germany). The proteins were diluted to 200 nM with PBS-T. As TraN and its variants featured an N-terminal 7×His-tag, a His-tag specific labeling method was used. The protein samples were mixed with RED-tris-NTA 2^nd^ Generation dye (NanoTemper Technologies GmbH) in PBS-T at a molar ratio of 2:1 to avoid unbound dye and were incubated in the dark for 30 min at RT. The fluorescently labeled proteins were centrifuged at 15,000 × g for 10 min. The supernatants were used for the binding assays.

The ds-oligonucleotides (oBS and random DNA, chapter 2.8) were diluted to 25.6 µM with PBS-T. A two-fold serial dilution of the oligonucleotides was performed with PBS-T in 500 µL reaction tubes as per the manufacturer’s manual (NanoTemper Technologies GmbH) resulting in final DNA concentrations of 12.8 µM to 0.781 nM (Supplementary Table S3). To prepare 15 different dilutions with DNA:protein ratios of 256:1 to 0.0156:1, the fluorescently-labeled protein samples (100 nM) were added to each dilution, resulting in a final concentration of 50 nM protein per sample. As a negative control, PBS-T buffer and 25 nM RED-tris-NTA 2^nd^ Generation dye was used. The samples were loaded into a capillary with a hydrophilic coating (NanoTemper Premium Capillaries, Cat# MO-K025, NanoTemper Technologies GmbH), placed into the capillary tray, and inserted into the Monolith NT.115 MST machine controlled by NTControl v2.1.31 software (NanoTemper Technologies GmbH). Capillary scans were performed at various LED power values before each measurement to ensure a sufficiently raw fluorescence signal (>200 counts). MST was performed at 60% LED and MST power with 5 s fluorescence detection before and after induction of local temperature gradient (Fluo before and Fluo after respectively) as well as with 30 s detection of the thermophoresis signal (MST On) and a 25 s delay between each measurement.

The data points were plotted using the MO. Affinity Analysis v.2.1.3 software (NanoTemper Technologies GmbH) and were fitted using the K_d_ model by setting the target concentration to 50 nM (final protein concentration). For calculation of the normalized fluorescence (F_norm_) value, the T-Jump + Thermophoresis preset with an automatically determined best signal-to-noise ratio was chosen. The curve fitting parameters including the K_d_ were estimated and automatically calculated by the software. The results are presented as plots of ΔF_norm_ against the ligand (dsDNA) concentration in nM.

### 2.10 Construction of plasmids for expression of pIP501 Tra proteins in *Priestia megaterium*

The *E. coli*/*P. megaterium* (previously known as *Bacillus megaterium*) shuttle vectors pRBBm59-RBS-*traN*-Strep and pRBBm59-RBS-*gfp*-Strep were constructed using Gibson Assembly (New England Biolabs). pRBBm59-RBS-*gfp*-Strep not encoding any pIP501 *tra* gene was used as a negative control in the pull-down assays. The backbone of pRBBm59-RBS-Strep was amplified using pRBBm59-RBS-*traB*-Strep (Berger et al., 2022) as a template using the primers GA_pRBBm59-RBS-Strep fw and GA_pRBBm59-RBS-Strep rev. The *traN* gene was amplified from pIP501 with GA_pRBBm59-traN fw and GA_pRBBm59-traN rev, and *gfp* was amplified from pRBBm59 by using the primers pRBBm59-GFP fw and pRBBm59-GFP rev (Supplementary Table S2). We chose *Priestia megaterium* as the expression host because it is a Gram-positive expression system and thus is better suited for a Gram-positive T4SS. Expression trials using *E. coli* hosts showed very low Tra protein expression. This approach has already been used recently by Berger et al. (2022).

*P. megaterium* MS941 (pMBGm19-RBS-*traB*-*traO*) (Berger et al., 2022) was transformed with pRBBm59-RBS-*traN*-Strep and pRBBm59-RBS-*gfp*-Strep, respectively, according to the protocol in Biedendieck et al. (2011). Transformants were selected on LB agar containing 35 μg/mL chloramphenicol and 10 μg/mL tetracycline and were screened for the presence of the respective pRBBm59 plasmids by colony PCR.

### 2.11 Expression and purification of Strep-tagged TraN and its interaction partners from *Priestia megaterium* MS941 (pMGBm19-RBS-*traB*-*traO*, pRBBm59-RBS-*traN*-Strep) lysate

Expression and purification of Strep-tagged TraN and its interaction partners from *P. megaterium* MS941 (pMGBm19-RBS-*traB*-*traO*, pRBBm59-RBS-*traN*-Strep) lysate were performed as per Berger et al. (2022) with some modifications.

The expression cultures (1.2 L in 5 L baffled flasks) were inoculated to an OD_600_ of 0.01 and incubated by shaking at 37°C to an OD_600_ of 0.3–0.4. The expression of TraB-TraO and Strep-tagged TraN was induced by the addition of 0.5% (w/v) xylose for pMGBm19 and 0.5% (w/v) sucrose for pRBBm59. After 5 hours, the cells were harvested by centrifugation at 9,000 × g at 4°C for 60 min. The cell pellet was resuspended in three times the cell wet weight of complex binding buffer (100 mM Tris/HCl, 300 mM NaCl, 1 mM EDTA, pH 8), supplemented with protease inhibitor (1 μg/mL pepstatin, 2 μg/mL antipain, 20 μg/mL leupeptin in DMSO). The cells were then lysed by sonication for 1 h on ice (50% duty cycle, 60% intensity; Sonopuls, UW/HD 2070; Bandelin electronic GmbH & Co. KG, Germany). After 60 min of centrifugation at 4°C and 38,000 × g, the supernatant was filtered through a MF-Millipore 0.45 µm MCE membrane (Merck, Darmstadt, Germany) to eliminate residual cell debris. The filtered lysate was loaded onto a 5-mL StrepTrap™ HP column pre-equilibrated with complex binding buffer using an ÄKTA pure 25-L chromatography system. The proteins were eluted using the complex binding buffer supplemented with 2.5 mM desthiobiotin. Peak fractions were loaded onto 12% SDS polyacrylamide gels and subjected to immunoblotting. Primary polyclonal anti-Tra antibodies were used against TraB, TraE, TraF, TraG, TraH, TraJ, TraK, TraM, and TraN (BioGenes GmbH) with a dilution of 1:10,000, and TraO with a dilution of 1:1,000, to probe for coeluted Tra proteins. We used as secondary antibody a horseradish peroxidase conjugated antibody against rabbit IgG (Promega GmbH), diluted to 1:10,000.

### 2.12 MS analysis of TraN-Strep-based pull-down assay

Bands containing the elution fractions of the pull-down were excised from a silver stained 12% SDS polyacrylamide gel and subjected to in-gel reduction, alkylation, and trypsinization as per Gesslbauer et al. (2018). Supplementary Table S6 shows the resulting peptides. Extracted peptides were purified and concentrated using C18 spin columns (Pierce™ C18 Spin Columns; Thermo Fisher Scientific) as per the manufacturer’s protocol.

The concentrated purified peptides were applied to liquid chromatography (LC) through an Ultimate 3,000 RSLCnano System (Thermo Fisher Scientific). Peptides were separated with a flow rate of 300 nL/min on a C18 Aurora UHPLC column (25 cm × 75 µm ID, 1.6 µm) with CaptiveSpray Insert (IonOpticks). The mobile phases were A) 0.1% (v/v) formic acid in water and B) 0.1% (v/v) formic acid in acetonitrile. The HPLC gradient for separation was 2% B for 6 min, 2%–25% B for 90 min, 25%–40% B for 10 min, 40%–80% B for 10 min, 80% B for 10 min, and 2% B for 12 min. The LC system was coupled online to a timsTOF Pro mass spectrometer (Bruker Corporation, Billerica, Massachusetts, United States) with a CaptiveSpray nano-electrospray ion source (Bruker); the mass spectrometer was operated in PASEF mode. TIMS, PASEF, and calibration settings were applied as described by Meier et al. (2018).

Mass spectrometry raw files were processed with MaxQuant version 2.0.1.0 (Cox and Mann, 2008). MS/MS spectra were searched against the UniProt *P*. *megaterium* (strain ATCC 12872/QMB1551) (*B*. *megaterium*) reference proteome databank (Proteome ID: UP000000935). The amino acid sequences of TraA, TraB, TraC, TraD, TraE, TraF, TraG, TraH, TraI, TraJ, TraK, TraL, TraM, TraN, and TraO were manually included in the *P. megaterium* FASTA file. Search parameters were set as follows: trypsin was set as enzyme; a maximum of two missed cleavages was allowed; the minimum peptide sequence length was seven amino acids; the maximum peptide mass was 4,600 Da. Carbamidomethylation of cysteine was set as a fixed modification; oxidation of methionine and acetylation of protein amino-termini were set as a variable modification. All other MaxQuant search parameters were equivalent to those described by Meier et al. (2018).

### 2.13 *In silico* search for TraN homologs in putative type IV secretion systems of Firmicutes

TraN homologs were identified with an iterative PSI-BLAST and tBLASTn search using the National Center of Biotechnology Information (NCBI) nucleotide database (https://www.ncbi.nlm.nih.gov/). The criteria selected were: 1) only putative functional proteins; 2) the presence of a putative *oriT* checked by oriTfinder server (https://bioinfo-mml.sjtu.edu.cn/oriTfinder/); 3) presence of a relaxase gene; 4) presence of an additional signature gene of conjugative plasmids; 5) only plasmid encoded TraN homologs; 6) exclusion of unnamed plasmid entries; 7) exclusion of artificial plasmids such as shuttle vectors. As all putative TraN homologs identified by an iterative PSI-BLAST using TraN as query were annotated as hypothetical proteins, we incorporated a tBLASTn search as an additional criterion for pre-selecting plasmid-encoded TraN homologs. The tBLASTn algorithm is used to compare protein query sequences against a nucleotide sequence database, allowing the detection of potential homologous DNA sequences that might not be annotated as protein.

The web server oriTfinder (Li et al., 2018) was used to rapidly detect putative *oriT*s and transfer-associated modules such as relaxase genes, type IV coupling protein (T4CP) genes, or T4SS gene clusters within the selected plasmid sequences. The *oriT* site is an essential module of self-transmissible elements. A multiple sequence alignment of selected TraN homologs was performed with T-Coffee (version 11.0) using the Expresso algorithm with default settings (Armougom et al., 2006; Di Tommaso et al., 2011). Additionally, all pIP501 transfer proteins (TraA to TraO) were selected as query sequences for tBLASTn searches to identify homologs in putative conjugative plasmids encoding *traN* homologous sequences.

PlasmidFinder 2.1 (Carattoli et al., 2014) was used to identify the incompatibility group of the plasmids selected according to the criteria above. FASTA-formatted plasmid DNA sequences from NCBI were uploaded to the website of the Center for Genomic Epidemiology (CGE; https://cge.food.dtu.dk/services/PlasmidFinder-2.0/) and analyzed using these settings: 75% minimum identity threshold; 60% minimum coverage threshold. The database for Gram-positive plasmid replicon sequences was selected. Replicon families/incompatibility groups of the plasmids were identified based on the detection of specific replication (*rep*) initiator genes. Plasmid sequences for which PlasmidFinder found no match were not further analyzed and were denoted “not characterized”.

### 2.14 Structural predictions of TraN and its homologs

The coLAB AlphaFold2 server (Mirdita et al., 2017; Mirdita et al., 2019; Mitchell et al., 2020; Jumper et al., 2021; Mirdita et al., 2022) was used for 3D structure prediction of TraN and its putative homologs. In addition, the structures of the TraN variants from pQTEV constructs (TraN, TraN_R23A-N24A-Q28A, TraN_G47A-G48A, TraN_H82A-R86A, and TraN_G100A-K101A) were predicted to check whether the amino acid substitutions had an influence on the protein fold. Further structural predictions were made for a set of conjugative transfer proteins from different Firmicutes conjugative plasmids (indicated in subscript) to identify shared secondary structure elements: TrsR_pUC11B_ (GenBank accession number: WP_032398615.1), TrsR_pUC08B_ (WP_032398615.1), TrsR_pAF22_ (WP_015062887.1), TrsR_p001F_ (WP_095348652.1), Tra20_pNP40_ (WP_012477765.1), TraR_pNP40_ (WP_012477778.1), Orf5_pMRC01_ (WP_010890662.1), and TrsR_pIBB477a_ (WP_010890662.1). To visualize and determine root mean square deviation (RMSD) values, we performed alignments of the predicted structures to the crystal structure of TraN_pIP501_ (PDB: 4P0Z, residues 1-117 are visible in the electron density) using PyMOL (PyMOL Molecular Graphics System, Version 2.6 Schrödinger, LLC). All alignments were performed using the “cealign” algorithm of PyMOL.

## 3 Results

### 3.1 pIP501 transfer is fully restored by complementing the *traN* knockout with pEU327-RBS-*traN*: single alanine substitutions of putative TraN key residues have only a minor effect on complementation

The transfer rate of the pIP501∆*traN* knockout has been shown to be two orders of magnitude higher than that of the original plasmid (Kohler et al., 2018a). However, this effect could not be restored to wild-type level by providing *traN* with the ribosomal binding site (RBS) of *traJ*_pIP501_ in pEU327 *in trans*. In this study, we constructed the complementation plasmid pEU327-RBS_*traN*_ *-traN* containing the original RBS of *traN*. Henceforth, pEU327-RBS_*traN*_-*traN* is referred to as pEU327-RBS-*traN*.

**FIGURE 1:**
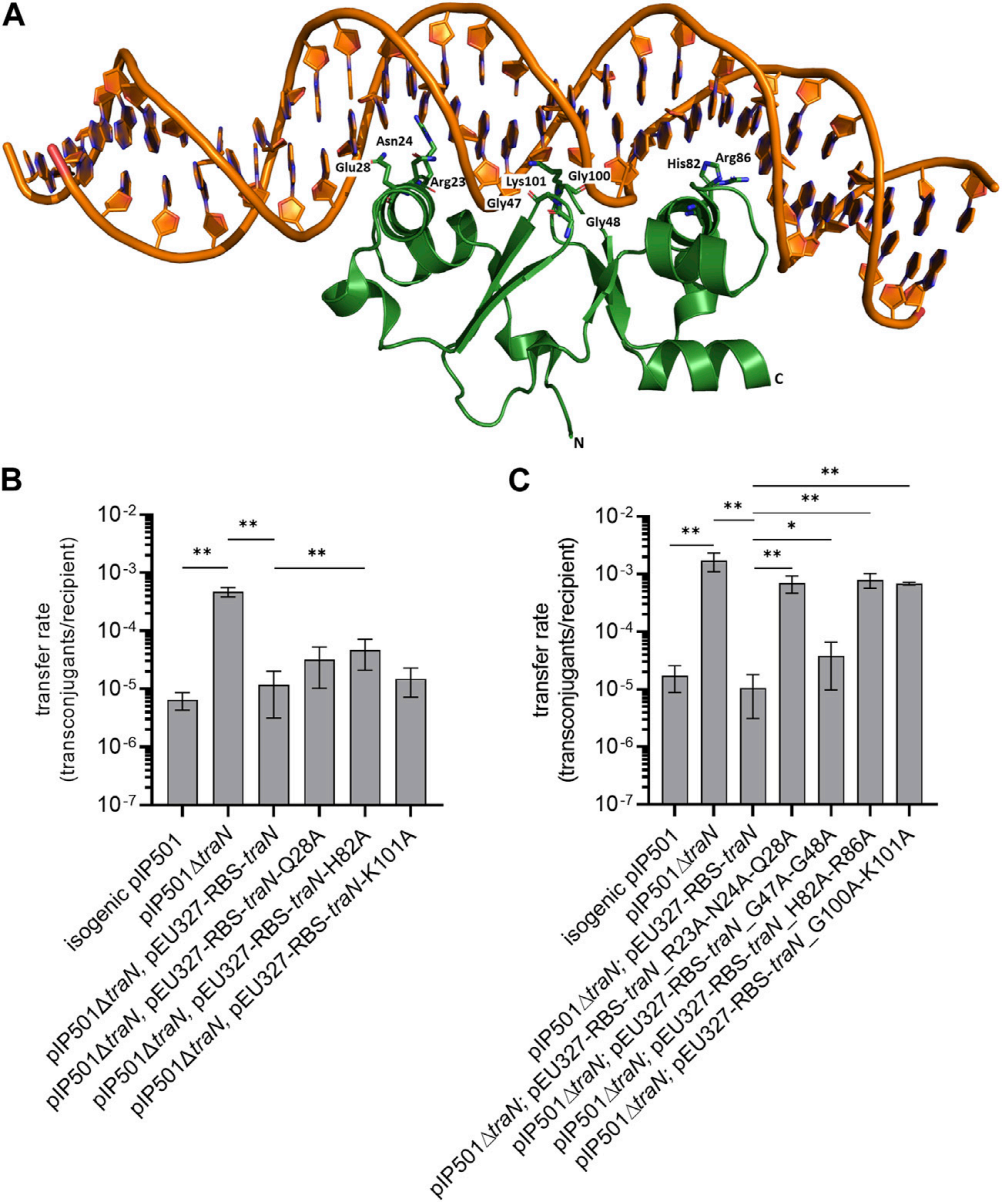
Biparental mating assays of pIP501, pIP501∆*traN*, and pIP501∆*traN* substituted with *traN* and *traN* carrying point mutations in the putative key residues listed below. *E. faecalis* JH2-2 (pIP501 isogenic wild type (obtained during generation of the *traN* knockout)), *E. faecalis* JH2-2 (pIP501∆*traN*) and *E. faecalis* JH2-2 (pIP501∆*traN* and pEU327-RBS-*traN*) were used as reference. **(A)** Crystal structure of TraN in complex (PDB: 6G1T) with original binding site (oBS). Mutated residues labeled and shown as sticks. **(B)** Complementation strains of *traN* knockout expressing single-substituted *traN* variants *in tran*s (pEU327-RBS-*traN*_Q28A, pEU327-RBS-*traN*_H82A, pEU327-RBS-*traN*_K101A) and **(C)** complementation strains of *traN* knockout expressing multiple-substituted *traN* variants *in trans* (pEU327-RBS-*traN*_R23A-N24A-Q28A, pEU327-RBS-*traN*_G47A-G48A, pEU327-RBS-*traN*_H82A-R86A, and pEU327-RBS-*traN*_G100A-K101A) were applied as donors, *E. faecalis* OG1X as recipient. Transfer frequencies are presented as number of transconjugants per recipient cell. n = 3. Mean values are depicted with standard deviation. **p < 0.01, *p < 0.1 as determined by the Mann–Whitney U test.

Providing pEU327-RBS-*traN in trans* to pIP501∆*traN* fully restored the wild-type pIP501 transfer rate in *E. faecalis* (Figures 1B,C). Thus, the polar effects of the *traN* knockout on the *traO* gene downstream in the *tra* operon can be excluded.

To investigate the impact of single alanine substitutions in TraN on pIP501 transfer, putative key residues were chosen based on their ability to form direct protein-to-base interactions which are defined as class 1 interactions (Kohler et al., 2018a) and direct protein-to-DNA-backbone interactions, respectively. Residues forming only water-mediated interactions were excluded. TraN key amino acid residues were identified based on the TraN/pIP501 DNA complex crystal structure (Kohler et al., 2018a) (Figure 1A). TraN contains two-winged helix-turn-helix (wHTH) motifs. Motif 1 is comprised of the amino acids interacting with the major groove of the DNA—residues R23, N24, and Q28—within the N-terminal TraN domain. Motif 2 is comprised of the residues H82 and R86 of the C-terminal domain, respectively. In addition, interactions with the minor groove of the DNA are exerted by the two wings of the wHTH motifs—residues G47–G48 and G100–K101 (Kohler et al., 2018a). To observe the effect of single alanine substitutions in TraN on pIP501 transfer, three amino acids were selected, each belonging to one DNA interaction site (Q28, H82, K101). Here, we exchanged these putative key residues against alanine by site-directed mutagenesis to disrupt the direct base contacts to the original TraN binding site upstream of the P_*tra*_ promoter of the pIP501 *tra* operon (Figure 1A). *In trans* complementation of pIP501∆*traN* was assayed with these variants cloned in the expression plasmid pEU327. Complementation effects were tested in biparental mating assays with *E. faecalis*.

Transfer rates of *E. faecalis* JH2-2 (pIP501∆*traN*) complementation strains (pEU327-RBS-*traN*_Q28A, pEU327-RBS-*traN*_H82A and pEU327-RBS-*traN*_K101A) were compared with pIP501, the complemented knockout with *traN in trans*, and the pIP501∆*traN* knockout strain. The single TraN amino acid substitution (H82A) revealed a small but significant increase in transfer rate (4.66 × 10^−5^) compared to complementation with *traN in trans* (1.17 × 10^−5^). Q28 and K101 substitutions led to non-significant changes in transfer compared to complementation with *traN* (Figure 1B). All transfer rates (mean values and standard deviations) are given in Supplementary Table S4.

### 3.2 Multiple substitutions of putative TraN key residues in the complementation plasmid led to a significant increase in pIP501∆*traN* transfer

As single amino acid substitutions of putative TraN key residues had only a minor effect on pIP501 transfer, three double alanine substitutions (G47A-G48A, H82A-R86A, G100A-K101A) and one triple substitution (R23A-N24A-Q28A) were generated. These residues are located on the DNA-binding α-helix and the wing of the wHTH motif of both N- and C-terminal DNA binding motifs. Variants of pEU327-RBS-*traN* with multiple alanine substitutions (G47A-G48A, R23A-N24A-Q28A, H82A-H86A, G100A-K101A) were constructed. *E. faecalis* JH2-2 (pIP501∆*traN*) was complemented with those variants. They were applied to biparental mating assays as donors and *E. faecalis* OG1X as recipient. Three of four multiple substitutions (R23A-N24A-Q28A, H82A-R86A, and G100A-K101A) revealed a significantly enhanced transfer rate. In each case, the differences were larger than one order of magnitude. The transfer rate was similar to that of the ∆*traN* knockout strain with a transfer rate of 1.72 × 10^−3^ (Figure 1C, Supplementary Table S4). Complementation of pIP501∆*traN* with *traN*-G47A-G48A showed the lowest effect (3.79 × 10^−5^) in comparison to complementation with *traN* (1.06 × 10^−5^) and the pIP501 transfer rate (1.72 × 10^−5^). Mean values and standard deviation of all transfer rates are given in Supplementary Table S4.

### 3.3 All TraN variants were expressed in *E. faecalis* JH2-2

To exclude the observed changes in transfer rate of pIP501∆*traN* complemented with *traN* variants cloned into pEU327 being due to lack of expression of the proteins, we verified their expression in *E. faecalis* JH2-2. Expression of TraN and seven TraN variants was investigated in *E. faecalis* JH2-2, harboring pIP501∆*traN* and pEU327 carrying *traN* or one of the *traN* variants.

TraN as well as all TraN variants were expressed in *E. faecalis* JH2-2 (Supplementary Figure S2). However, the level of expression appeared to differ. TraN_H82A-R86A and TraN_G100A-K101A particularly showed lower expression than TraN and the other variants (Supplementary Figure S2), so we verified whether the alanine substitutions changed the binding affinity of the anti-TraN antibody. The four multiple-substituted TraN variants and TraN (TraN, TraN_R23A-N24A-Q28A, TraN_G47A-G48A, TraN_H82A-R86, and TraN_G100A-K101A) were expressed in *E. coli* Lemo21 (DE3), purified as described in chapter 2.6 and applied to immunoblotting (Supplementary Figure S1C), resulting in signals with differing intensity for TraN and the variants.

Especially TraN_H82A-R86A, with the lowest signal intensity in the immunoblot checking the expression (Supplementary Figure S2), and also TraN_R23A-N24A-Q28A, and TraN_G100A-K101A yielded a weaker signal in the immunoblot than TraN. As we were using a polyclonal anti-TraN antibody, we assumed that it binds to different TraN epitopes. If one of these epitopes is changed due to alanine substitution(s), it may result in a weaker signal in the immunoblot. Thus, it can be argued that the lower signal intensity for these TraN variants in *E. faecalis* might be due to the mutation(s) of epitopes recognized by anti-TraN antibodies and not due to lower expression levels. In addition, we want to emphasize that, due to application of the overexpression plasmid pEU327, sufficient protein levels of the TraN variants TraN_H82A and TraN_H82A-R86 will be present, although expression levels of these two TraN variants seem to be lower—as evidenced in the immunoblot (Supplementary Figure S2).

### 3.4 Expression and purification of TraN and its variants resulted in similar yields of pure protein

**FIGURE 2:**
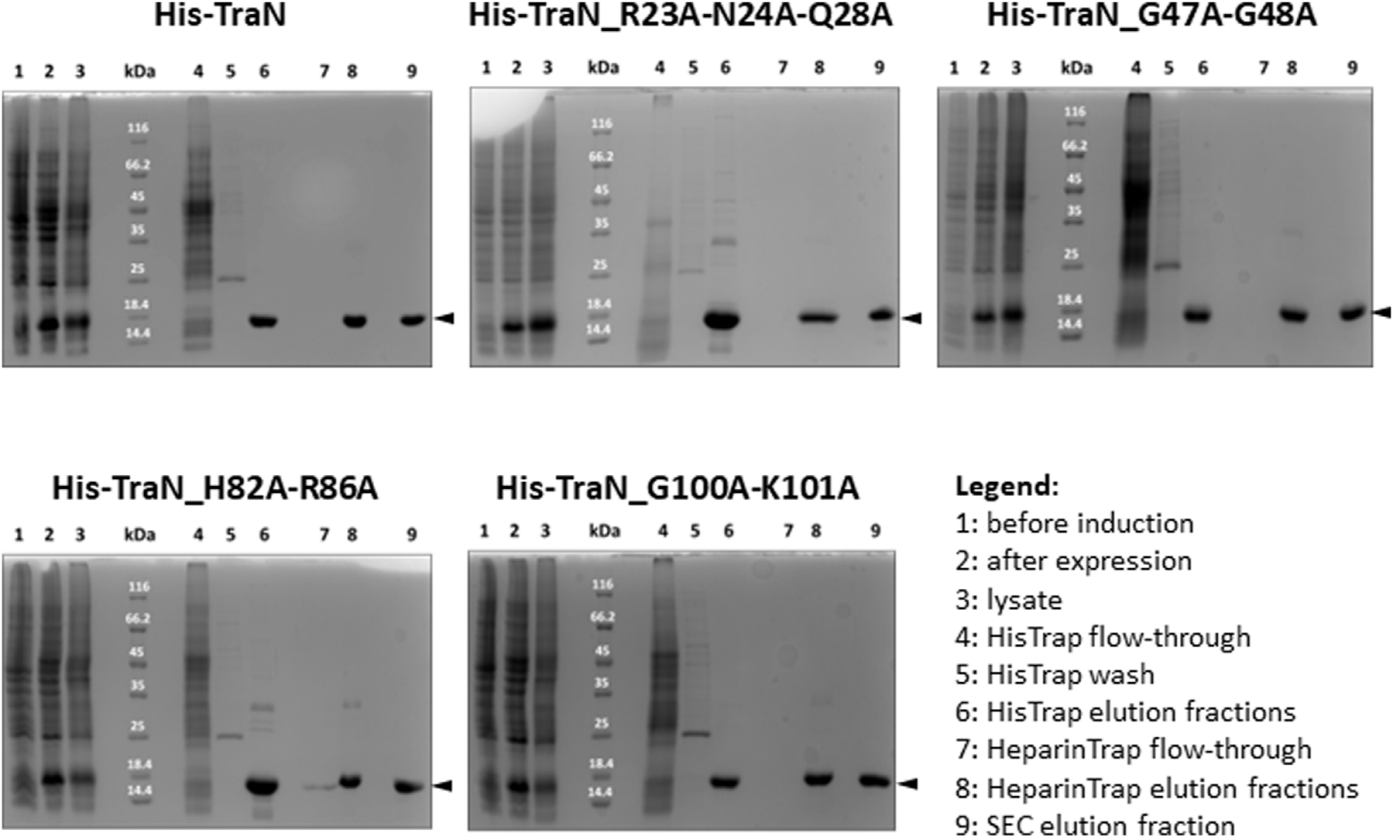
Coomassie-stained 12% SDS polyacrylamide gels of expression and purification of TraN and its variants. kDa: Pierce™ Unstained Protein molecular weight marker (Thermo Fisher). The expected molecular weight of all constructs ranges from 17.10 kDa (TraN_R23A-N24A-Q28A) to 17.32 kDa (TraN_G47A-G48A).

TraN and the four variants (TraN_R23A-N24A-Q28A, TraN_G47A-G48A, TraN_H82A-R86A, TraN_G100A-K101A) were expressed in *E. coli* Lemo21 (DE3) and purified by HisTrap, followed by HeparinTrap affinity chromatography. To assess purity and oligomerization states, an analytical size-exclusion chromatography (SEC) was performed. All variants were well expressed and exhibited similar final yields of purified proteins. Corresponding SDS polyacrylamide gels of all purification steps are shown in Figure 2 and the chromatograms showing the elution profile of purified TraN and its variants in Supplementary Figure S1B. TraN and its variants showed a pronounced overexpression comparing the samples before induction with the samples at harvesting. The elution fractions of the analytical SEC runs showed that we retrieved TraN and all its variants in high purity. The 260/280 ratio indicates that TraN and its variants were all free from bound DNA, which is a crucial prerequisite for DNA binding studies (Supplementary Figure S1B).

### 3.5 Amino acid substitutions in TraN do not impair the overall fold of the protein

**FIGURE 3:**
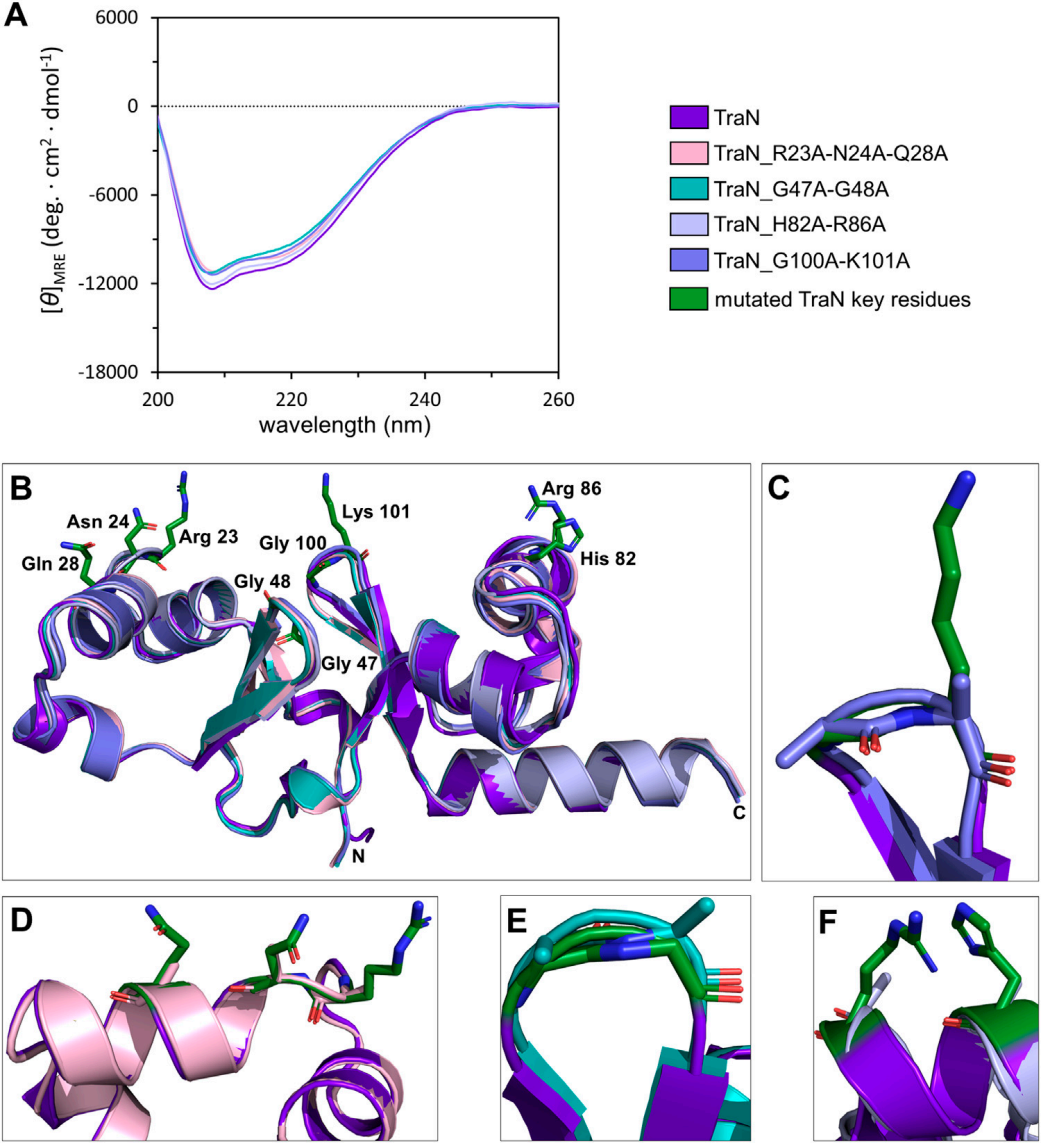
TraN fold seems unaffected in TraN variants, TraN_R23A-N24A-Q28A, TraN_G47A-G48A, TraN_H82A-R86A, and TraN_G100A-K101A. **(A)** Circular dichroism (CD) spectra of His-TraN and its variants. **(B)** Structural alignment of the crystal structure of TraN and the predicted structures of its variants showing that the substitutions do not have an impact on the protein fold. TraN is depicted in purple. The residues mutated in the variants are shown as green sticks. C- and N-termini are labeled. The color code for the TraN variants is shown in Supplementary Table S5. **(C)** Close-up of the wing motif of the C-terminal wHTH motif (TraN_G100A-K101A). The alanines likely protrude into the minor groove upon DNA binding. **(D)** Close-up of motif 1. The mutated residues of TraN_R23A-N24A-Q28A are shown as light pink sticks. **(E)** Close-up of the wing motif of the N-terminal wHTH motif (TraN_G47A-G48A). Introduced alanine residues likely protrude into the minor groove upon DNA binding. **(F)** Close-up of motif 2 (TraN_H82A-R86A).

Circular dichroism (CD) measurements were performed to compare whether multiple alanine substitutions affect the fold and stability of TraN. All TraN variants showed almost identical CD spectra compared to TraN, displaying two minima at 207 nm and 216 nm. This suggests that there are no significant changes in secondary structure between the variants (Figure 3A).

We used the CoLab AlphaFold2 server for structure prediction of the TraN variants (TraN_Q28A, TraN_H82A, TraN_K101A, TraN_H82A-R86A, TraN_G47A-G48A, TraN_G100A-K101A, TraN_R23A-N24A-Q28A). The alignment of multiple-substituted TraN variants with the experimental TraN structure resulted in overall RMSD values between 0.588 Å (TraN_G47A-G48A) and 0.643 Å (TraN_H82A-R86A). The alignment of single-substituted variants to TraN gave even lower RMSD values of 0.519 Å to 0.540 Å (Supplementary Table S5). Alignments of the double- and triple-substituted variants are shown in Figure 3B. In addition, we provide close-up images of the regions showing the mutated residues (Figures 3C–F). The low RMSD values indicate that the mutations do not affect the protein fold in the predicted structures. In comparison, the alignment of the crystal structures of free TraN (PDB: 4P0Z) with TraN in complex with oBS (PDB: 6G1T) yielded a RMSD value of 0.837 Å.

### 3.6 DNA binding affinities of TraN variants correlate with respective pIP501 transfer rates

**FIGURE 4:**
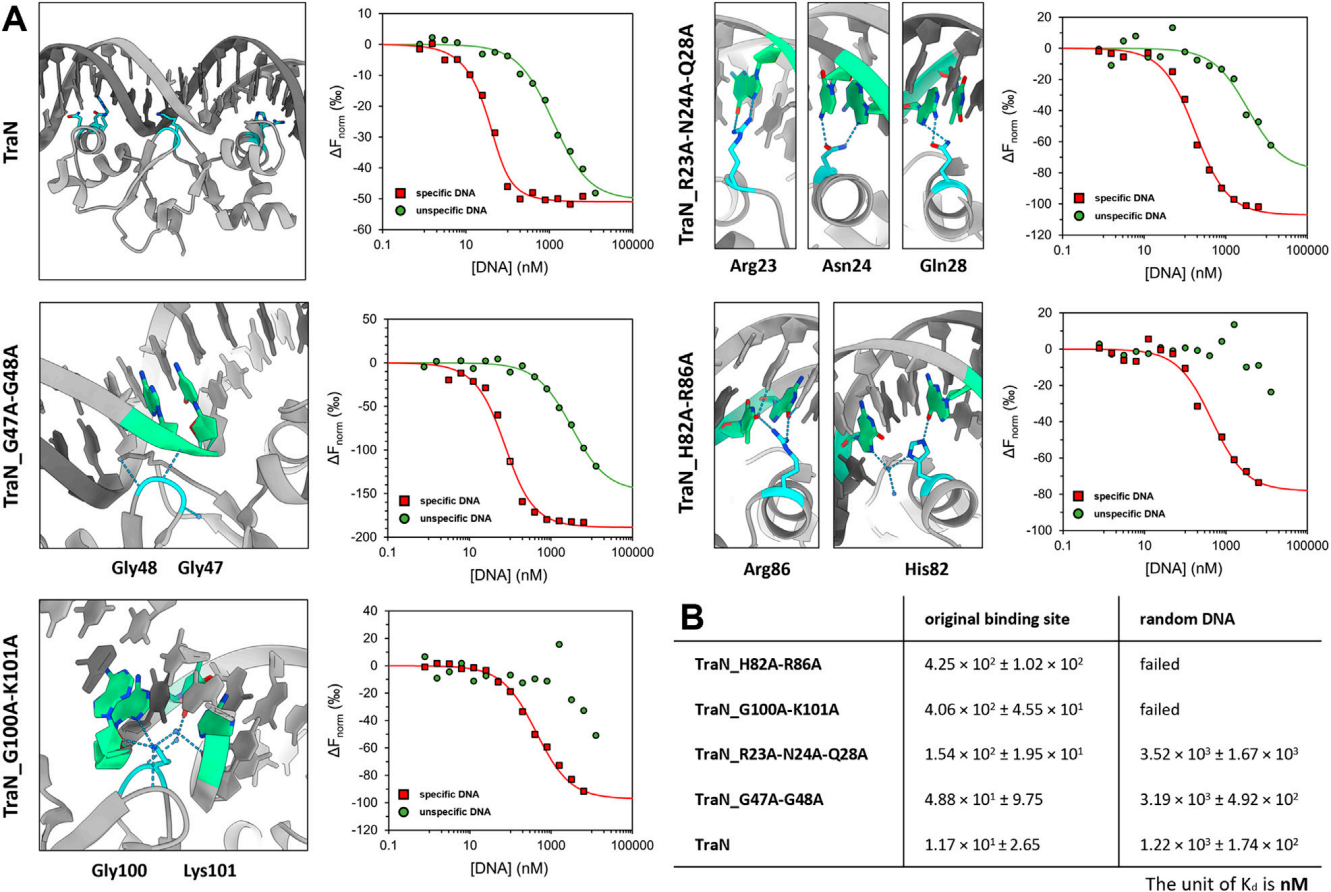
Graphical representation of TraN and its variants interacting with original binding site (oBS) and random ds-oligonucleotide (random DNA). **(A)** Cartoon representation of class (I) interactions between amino acid side chains and corresponding bases of oBS. The images were taken from the structure of TraN bound to oBS pIP501 DNA (PDB: 6G1T). Interacting residues are highlighted in cyan and respective bases of the oBS are highlighted in green. Water molecules are depicted in dark blue, hydrogen bonds in light blue. Normalized dose-response curves of TraN and its variants for interactions with oBS (red) and unspecific random DNA (green) acquired from MST binding assays. Wherever the fit was successful, the resulting curves exhibit a sigmoidal shape from which a dissociation constant (K_d_) was calculated. **(B)** MST-derived dissociation constants (K_d_) of TraN and its variants to oBS and random DNA.

To investigate the sequence-specific binding affinities of the TraN variants (TraN, TraN_R23A-N24A_Q28A, TraN_G47A-G48A, TraN_H82A-R86A, and TraN_G100A-K101A), we used MST with dsDNA substrates corresponding to the original TraN binding site (oBS) upstream of the pIP501 *tra* operon, and a random pIP501 sequence of the same size (34-mer). The oligonucleotides are identical to the 34-bp dsDNA (oBS) identified and used by Gössweiner-Mohr et al. (2014a) for isothermal titration calorimetry (ITC) measurements. Dose-response curves of all protein-DNA combinations are shown in Figure 4A. Additionally, the sites where mutations were introduced are shown on the TraN-DNA complex crystal structure (PDB: 6G1T) (Kohler et al., 2018a). Close-up images of the mutated sites are also depicted in Figure 3. The dissociation constants for the interaction of TraN and its variants with the ds-oligonucleotides are shown in Figure 4B. All fitted curves exhibit a sigmoidal shape from which the K_d_ was calculated. It was not possible for the interaction of the double-substituted proteins TraN_H82A-R86A and TraN_G100A-K101A with the random DNA to fit the data points by a sigmoidal curve, suggesting an extremely low binding affinity. In the presence of the random DNA, the K_d_ values for all other TraN variants were in the low micromolar range, suggesting a much lower affinity than the binding of the specific 34-mer (oBS).

TraN showed the strongest binding affinity to the oBS 34-mer sequence (1.17 × 10^1^ ± 2.65 nM) followed by the double-substituted variant TraN_G47A-G48A (4.88 × 10^1^ ± 9.75 nM). The triple-substituted variant TraN_R23A-N24A-Q28A showed an almost 13-fold increase in the K_d_ value (1.54 × 10^2^ ± 1.95 × 10^1^ nM) compared to TraN, suggesting a strong negative effect on DNA binding affinity. Both TraN_H82A-R86A (4.25 × 10^2^ ± 1.02 × 10^2^ nM) and TraN_G100A-K101A (4.06 × 10^2^ ± 4.55 × 10^1^ nM) variants showed approximately 36-fold higher K_d_ values yielding the lowest binding affinities to the oBS 34-mer of all variants compared to TraN.

The binding affinities determined through MST exhibit a strong correlation with the results obtained from the biparental mating assays. When the pIP501∆*traN* knockout strain was complemented with *traN_*G47A-G48A *in trans* (3.79 × 10^−5^ transconjugants/recipient), the impact on pIP501 transfer was minimal compared to complementation with *traN*-G100A-K101A (6.86 × 10^−4^) (Figure 1B). Consequently, the binding affinity of TraN_G47A-G48A was found to be higher than that of TraN_G100A-K101A, indicating its strongest capability in sequence-specific binding on pIP501 oBS amongst the tested variants.

### 3.7 TraN pull-down reveals interactions with other pIP501 transfer proteins

Previously, yeast two-hybrid studies showed interactions of TraN with TraE, TraG, TraH, and TraL (Abajy et al., 2007). Therefore, we decided to perform a pull-down assay i) to confirm these interactions and ii) to elucidate further direct or indirect TraN interactions. Consequently, *P. megaterium* MS941 was co-transformed with pMGBm19-RBS-*traB*-*traO* and pRBBm59-RBS-*traN*-Strep. This resulted in co-expression of C-terminally Strep-tagged TraN (TraN-Strep) and pIP501 transfer proteins TraB to TraO in *P. megaterium* MS941.

Utilizing affinity chromatography, Strep-tagged TraN and the proteins interacting with it were purified from the clarified supernatant of *P. megaterium* MS941 (pMGBm19-RBS-*traB*-*traO*, pRBBm59-RBS-*traN*-Strep) lysate.

**FIGURE 5:**
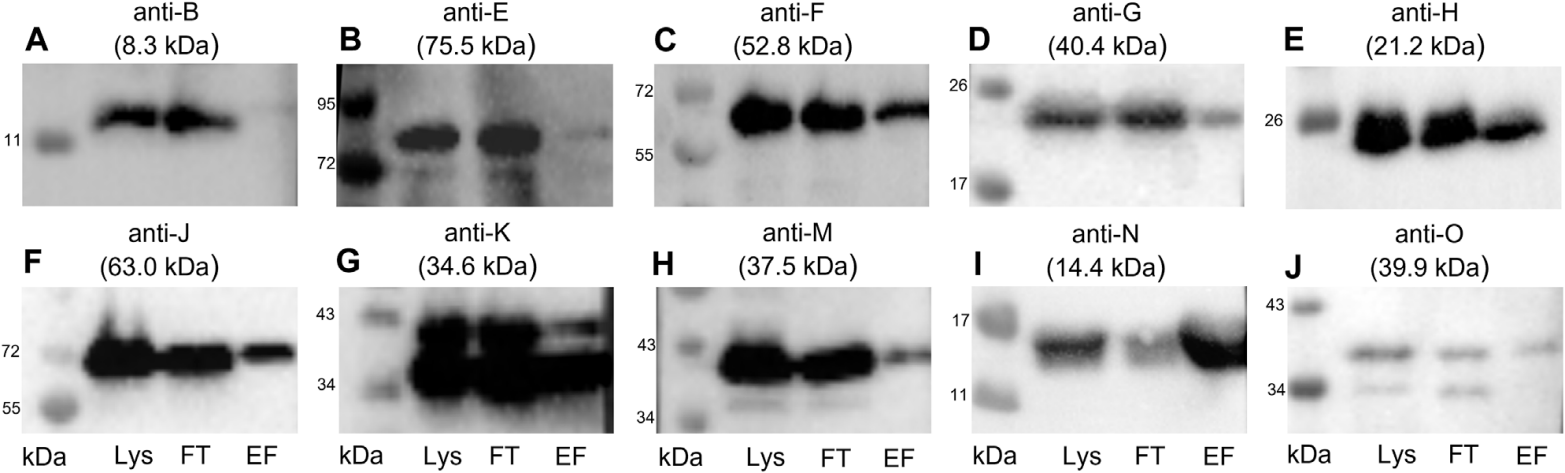
Western blots of the elution fractions of the pull-down with TraN-Strep. The following antibodies were used: **(A)** anti-TraB, **(B)** anti-TraE, **(C)** anti-TraF, **(D)** anti-TraG, **(E)** anti-TraH, **(F)** anti-TraJ, **(G)** anti-TraK, **(H)** anti-TraM, **(I)** anti-TraN, and **(J)** anti-TraO, targeting the respective Tra proteins. The MW of the respective protein is indicated above each blot. MW marker (Blue Prestained Protein Standard, New England Biolabs) was included to assess protein sizes (kDa). Three fractions of the TraN-based pull-down were analyzed: lysate (Lys) in lane 1, flow-through (FT) in lane 2, and elution fraction (EF) in lane 3. *14.4 kDa is the MW of native TraN. The fused Strep-tag adds 1 kDa; therefore, the protein appears slightly larger than in the immunoblots performed with native TraN.

To detect coeluted Tra proteins, elution fractions from TraN pull-downs were subjected to immunoblotting with anti-Tra antibodies directed against TraB, TraE, TraF, TraG, TraH, TraJ, TraK, TraM, TraN, and TraO. The elution fraction was compared to the flow-through and lysate fraction. We could demonstrate the coelution of TraE, TraF, TraG, TraH, TraJ, TraK, TraM, and TraO, as evidenced by the presence of the respective bands (Figures 5A–J).

TraB was not coeluted, suggesting no interaction with TraN. In contrast with the calculated molecular weight (MW) of 8.3 kDa, the expected MW on the SDS polyacrylamide gel and the corresponding immunoblot is around 12 kDa (Berger et al., 2022). The membrane incubated with anti-TraO antiserum showed two bands which most likely present two differently processed forms of the protein: one with its signal sequence and one without it. The cleavage results in a size difference of 2.5 kDa (Berger et al., 2022). The single band representing TraG had a MW of around 20 kDa instead of the calculated MW of 40.4 kDa. It is hypothesized that the T4SS protein complex includes only the CHAP domain of TraG, as the observed band corresponds to its calculated MW of 22 kDa (Arends et al., 2013; Berger et al., 2022). Likewise, TraF was detected at around 60 kDa, which was slightly above the calculated 52.8 kDa. TraE was coeluted in rather small amounts. The nitrocellulose membrane incubated with the anti-TraK antibody showed two bands (Figure 5G), indicating the presence of two TraK variants. This can be attributed to the existence of two different translational start codons for TraK (Goessweiner-Mohr et al., 2014b). The coelution of TraM might be because TraN and TraM are both DNA binding proteins. In contrast to its C-terminal domain, the N-terminal domain of TraM showed unspecific DNA binding (T. Berger and W. Keller, unpublished data).

**FIGURE 6:**
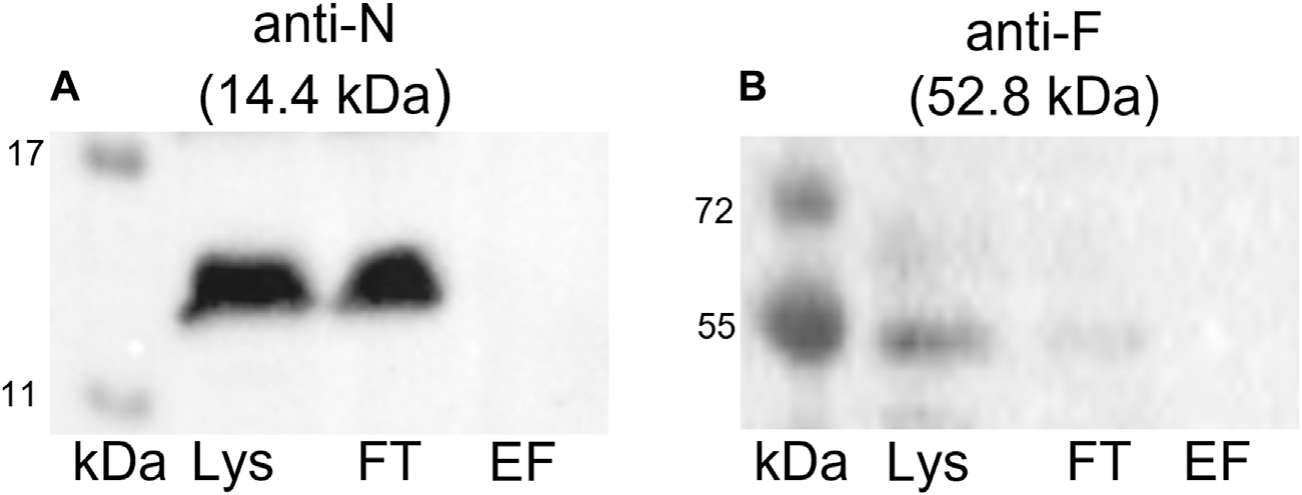
Western blots of the pull-down with GFP-Strep. The cell lysate (Lys) in lane 1, the flow-through (FT) in lane 2, and the elution fraction (EF) in lane 3 were analyzed. **(A)** Anti-TraN and **(B)** anti-TraF antibodies were used as primary antibodies. The used antibody and the MW of the respective Tra protein are given above each blot. MW marker (kDa) (Blue Prestained Protein Standard, New England Biolabs) was applied.

To confirm that the coelution of proteins together with the Strep-tagged TraN is highly specific and not the result of unspecific binding, we conducted a negative control experiment. A GFP pull-down was performed followed by immunoblotting using anti-TraN or anti-TraF antibodies (e.g., for a Tra protein which was coeluted in the TraN pull-down). TraN and TraF could be detected only in the lysate and flow-through fraction but not in the elution fraction (Figure 6), suggesting that there is no unspecific binding of pIP501 proteins.

Next, we analyzed the elution fractions from TraN pull-downs with high-resolution mass spectrometry. An in-gel tryptic digest was performed from the elution fractions, and the resulting peptides were analyzed by nano-LC-MS/MS. For the pull-down elution fractions, the MS spectra yielded positive hits for all Tra proteins except for TraA (not included in the expression vector, negative control), TraC, TraG, TraL, and TraO. Looking at the LFQ intensity signals of the samples (blue labels in Supplementary Table S7), TraE, F, H, J, K, M, and N show strong signals. TraN occurs in a huge surplus over the other Tra proteins as it carries the Strep-tag and binds to the affinity column whether attached to other Tra proteins or as a monomeric protein. Notably, TraB and TraD are detected but not clearly quantifiable, indicating their low abundance in the pull-down fraction.

### 3.8 TraN homologs are mainly encoded on putative conjugative Inc18 plasmids

Homology searches with TraN were performed to find putative similar proteins in other bacterial species or even genera.

A previous search for TraN homologs was performed by Kohler et al. (2018a). Despite their common presence in mobile genetic elements from Firmicutes, TraN homologs have been rarely investigated. As many new plasmid sequences have been submitted to NCBI in the last 5 years, we conducted a new search for putative TraN homologs. Additionally, we not only used a larger dataset but also assigned the selected plasmid sequences to putative conjugative and non-conjugative plasmids. According to the criteria defined in chapter 2.12, 54 plasmid sequences and one transposon sequence were selected for further analysis. All protein sequences displaying over 87% identity on amino acid level to TraN originated from Firmicutes.

According to the tBLASTn alignments, 26 out of 54 homologs displayed 100% identity on protein level with TraN, and 39 out of 54 homologs exhibited 100% query coverage with protein identities between 88% and 99% (Supplementary Table S8).

An *oriT* was predicted for 44 out of the 54 plasmids (see Supplementary Table S8). For the remaining ten, a tBLASTn search with the sequence of the pIP501 relaxase TraA, the ATPase TraE, and the coupling protein TraJ also revealed no hits. A putative TraJ_pIP501_ homolog was identified for the *oriT*-lacking plasmids pSM19035 (AY357120.1), pF104_1 (CP072892.1), pEGM181-1 (CP050484.1), and pGMrib (CP045673.1). Plasmid pEGM181-1 additionally encodes for a TraE_pIP501_ homolog but not for a TraA_pIP501_ (Supplementary Table S9). Among the 54 plasmids with a predicted *oriT*, p2 (CP041740.1), plas1 (CP064340.1), and pVEF3 (AM931300.1) lack *traA*-/*traE*-/*traJ*-like sequences, with p2 being the only one encoding for a TraJ_pIP501_ homolog (Supplementary Table S9).

Supplementary Table S8 provides additional information on the presence of a pIP501-like T4SS for the initially selected 54 plasmid sequences and one transposon sequence. The NCBI tBLASTn search employed the pIP501 transfer proteins TraA to TraO as queries. Of these MGEs, 65% encode a complete T4SS (*traA*—*traO*), underlining the conjugative potential of the TraN-encoding MGEs. Interestingly, four putative conjugative plasmids (p3-38, pEFS108_1, pL15-B, pW3) were identified as having a nearly complete T4SS cluster, lacking only a *traO*-like gene (Supplementary Table S8).

In total, at an amino acid level, 11 different TraN sequences were detected in 28 different plasmids.

**FIGURE 7:**
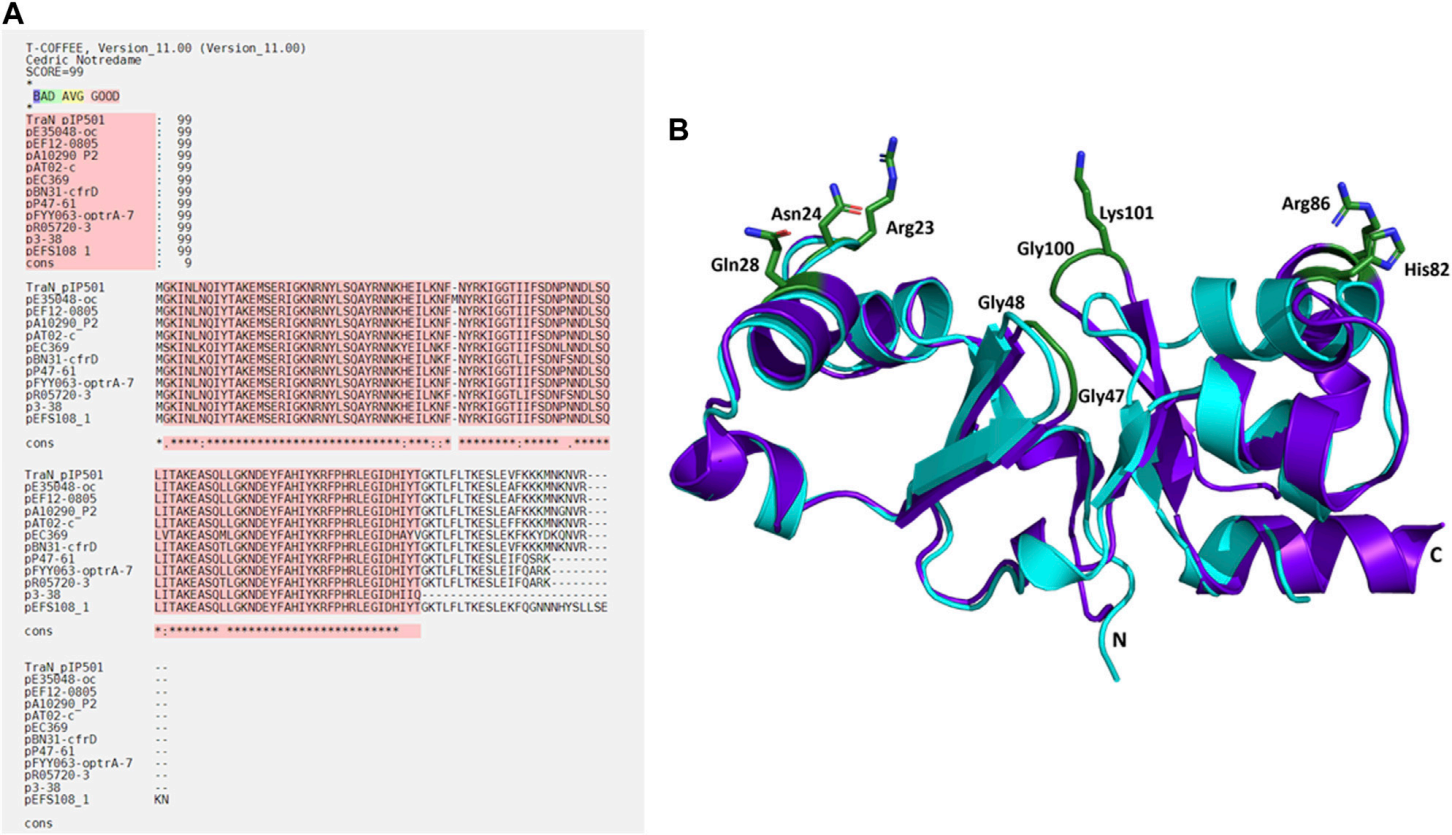
Alignments of TraN_pIP501_ and its homologs. **(A)** Multiple sequence alignment using Expresso algorithm implemented in T-Coffee. Homologs were identified by tBLASTn search. As these homologs are hypothetical, the names of the plasmids encoding the TraN_pIP501_ homolog are used instead of protein annotation. Asterisks (*) represent conserved amino acids in all aligned sequences, (:) indicates amino acids of high similarity, dots (.) indicate partial similarities. The top panel shows the evaluation scores from the T-Coffee Server (TCS) evaluation. The closer the score to 100, the better the alignment. The quality of the alignment is also represented in red. **(B)** Backbone alignment of TraN_pIP501_ (purple) with the homolog from p3-38 (cyan) using theoretical structures predicted with coLAB AlphaFold2 server. Residues (Arg23, Asn24, Gln28, Gly47, Gly48, His82, Arg86, Gly100, and Lys101) are shown as green sticks.

Eleven plasmids, each representing a distinct TraN_pIP501_ homolog and containing a putative *oriT*, were selected from the initial set of 28 (Table 1). Each TraN homolog shares ≤99% protein identity with TraN_pIP501_. Table 1 displays homologs of three pIP501 signature proteins—TraA_pIP501_, TraE_pIP501_, and TraJ_pIP501_—in the 11 selected plasmid sequences, indicating the likely presence of a T4SS in these plasmids that encode *traN* homologous sequences. All plasmids encoding a TraN_pIP501_ homolog exhibited high-level protein identity (≥96%) to the pIP501 TraA, TraE, and TraJ T4SS proteins (Table 1). The 11 different TraN homologs were applied to a multiple sequence alignment using T-Coffee Expresso (Figure 7A).

**TABLE 1:** TraA_pIP501_, TraE_pIP501_, and TraJ_pIP501_ homologs in putative conjugative plasmids encoding *traN* homologous sequences. All plasmids have a putative *oriT* predicted with oriTfinder.

Plasmid origin	aa identity with TraN [%]	T4SS_pIP501_ homologs	Protein identity [%]
pIP501^1,2^	TraN_1-122_	TraA_1-661_^a^	-
		TraE_1-653_^b^	-
		TraJ_1-551_^c^	-
pE35048-oc^3^	98 (TraN_1-122_)	MobA	96 (TraA_1-661_)
		TrsE	98 (TraE_1-653_)
		TrsK	99 (TraJ_1-551_)
pEF12-0805^4^	99 (TraN_1-122_)	MobA/MobL family	99 (TraA_1-204_), 89 (^d^TraA_206-661_)*
		TrsE	98 (TraE_1-653_)
		TrsK	99 (TraJ_1-551_)
pP47-61^5^	97 (TraN_1-114_)	MobA/MobL family	98 (TraA_1-661_)
		TrsE	98 (TraE_1-653_)
		T4SS conjugative DNA transfer family	99 (TraJ_1-551_)
pAT02-c^6^	99 (TraN_1-122_)	MobA/MobL family	98 (TraA_1-661_)
		TrsE	98 (TraE_1-653_)
		T4SS conjugative DNA transfer family	99 (TraJ_1-551_)
pEC369^7^	88 (TraN_1-122_)	MobA	98 (TraA_1-661_)
		TrsE	97 (TraE_1-653_)
		Conjugal transfer protein	98 (TraJ_1-551_)
pBN31-cfrD^8^	94 (TraN_1-122_)	MobA/MobL family	96 (TraA_1-661_)
		TrsE	98 (TraE_1-653_)
		T4SS conjugative DNA transfer family	99 (TraJ_1-551_)
pFYY063-optrA-70K^9^	97 (TraN_1-114_)	MobA/MobL family	98 (TraA_1-661_)
		TrsE	98 (TraE_1-653_)
		T4SS conjugative DNA transfer family	98 (TraJ_1-551_
pR05720-3^10^	91 (TraN_1-114_)	MobA/MobL family	96 (TraA_1-661_)
		TrsE	98 (TraE_1-653_)
		T4SS conjugative DNA transfer family	99 (TraJ_1-551_)
p3-38^11^	97 (TraN_1-97_)	MobA/MobL family	96 (TraA_1-661_)
		TrsE	98 (TraE_1-653_)
		TrsK	99 (TraJ_1-551_)
pEFS108_1^12^	98 (TraN_1-111_)	MobA/MobL family	98 (TraA_1-661_)
		TrsE	99 (TraE_1-653_)
		T4SS conjugative DNA transfer family	99 (TraJ_1-551_)
PA10290_P2^14^	98 (TraN_1-122_)	MobA/MobL family	96 (TraA_1-661_)
		TrsE	98 (TraE_1-653_)
		T4SS conjugative DNA transfer family	98 (TraJ_1-551_)

Names in the third column, T4SS_pIP501_ homologs are those found in the annotation of these plasmids in the NCBI database. GenBank accession numbers:^1^ AJ505823.1,^2^ L39769.1,^3^ MF580438.1,^4^ KY579372.1,^5^ CP091102.1,^6^ CP097064.1,^7^ CP032740.1,^8^ CP081834.1,^9^ CP116030.1,^10^ CP064409.1,^11^ JQ911740.1,^12^ CP085295.1,^13^ JQ911741.1, and ^14^ CP059757.1. Three pIP501 transfer proteins were selected for protein homology searches: ^a^ the relaxase TraA (number of amino acids in subscript) (AAA99466.1), ^b^ the ATPase, TraE (number of amino acids in subscript) (AAA99470.1), ^c^ the coupling protein TraJ (number of amino acids in subscript) (CAD44390.1), ^d^ sequence position (KY579372.1) 64,688–66,055 bp. *Two TraA-like segments are given because the *traA*-like gene is disrupted by an IS*605-*like element (ARQ19352.1). Percentage protein identity based on the BLAST algorithm is given.

#### 3.8.1 TraN homologs are also encoded on RepA_N family and Rep_3 family plasmids

The PlasmidFinder tool was used for *in silico* prediction of plasmid replicon families of all 54 plasmids. The affiliations of the plasmids to a replicon family and their corresponding identity scores are listed in Supplementary Table S8. Of the 54 plasmids, 43 were assigned as Inc18 broad-host-range plasmids, three plasmids belonged to the RepA_N family, and one to the Rep_3 family. No replicon sequence matches were found for seven of the plasmids. TraN homologs were found within the T4SS region of putative conjugative plasmids belonging to Rep_3 and RepA_N incompatibility groups, including plasmids like pL9 (Rep_3) and pL15-B (RepA_N), with pL15-B lacking a *traO*-like gene. In essence, our findings reveal the presence of TraN across diverse plasmid replicon families, extending beyond Inc18. While *traN*, together with other putative T4SS genes, has been identified in many plasmids, conjugative transfer has been demonstrated experimentally for a only few, underlining the limited knowledge of Inc18 plasmids.

To the best of our knowledge, conjugative plasmid transfer has only been demonstrated for 11 of these plasmids: pRE25 (Schwarz et al., 2001), pAMβ1 (Clewell et al., 1974), pV386 (Cinthi et al., 2022), pPPM1000 (Davis et al., 2005), pWZ909 (Zhu et al., 2010), pW3, p3-38 (Liu et al., 2013), pBN31-*cfrD* (Zhu et al., 2022), pEC369 (Yin et al., 2018), pEFS108_1 (Kim et al., 2021), and pP47-61 (Li et al., 2022). Conjugation experiments using donor strains with pE34048-oc (Morroni et al., 2018), pEF12-0805 (Lazaris et al., 2017), and pStrcfr (Wang et al., 2013) failed. pSM19035 is annotated as non-conjugative (Behnke et al., 1981). Interestingly, an additional TraN homolog was found on the composite transposon Tn*6349* located on the multiresistant *Staphylococcus aureus* strain AOUC-0915 (D’Andrea et al., 2019) encoding for *erm*(B), *cfr*, *poxtA*, and *fexB* resistance genes. However, the transposon was shown to be non-conjugative (D'Andrea et al., 2019).

### 3.9 TraN_pIP501_ key residues are not affected in TraN homologs

Sequence alignments with T-Coffee Expresso were performed to determine the extent to which TraN homologous sequences deviate from TraN_pIP501_. As depicted in Table 1, five TraN homologs are truncated: that encoded on p3-38 (TraN_1-97_), the homolog from pEFS108_1 (TraN_1-111_), and those from pP47-61, pFYY063-optrA-70K and pR05720-3 (TraN_1-114_). The coLAB AlphaFold2 server was used to predict the structures of the 11 distinct TraN_pIP501_ homologs. The structures were aligned with the solved structure of TraN (PDB: 4P0Z). As the sequence identity is quite high, the overall structure of all homologs is likely very similar to TraN_pIP501_. The respective RMSD values are listed in Table 2. The most notable difference is observed in the homolog encoded on p3-38 (Figure 7B), where not all key residues are conserved. This discrepancy is attributed to its smaller size (99 aa instead of 122 aa), leading to the absence of Gly100 and Lys101. In all other homologs, the corresponding amino acids (Arg23, Asn24, Gln28, Gly47, Gly48, His82, Arg86, Gly100, and Lys101) were not altered, indicating that the mode of DNA recognition is conserved among all homologs compared.

**TABLE 2:** TraN homologs aligned with the crystal structure of TraN_pIP501_ (PDB: 4P0Z) showing the root mean square deviation (RMSD) values for the backbone atoms. The number of the aligned amino acids is included.

Plasmid encoding a TraN_pIP501_ homolog	RMSD value [Å]	Number of aligned C_α_ atoms
pE35048-oc	0.983	112
pEF12-0805	0.644	112
pA10290_P2	0.569	112
pAT02-c	0.621	112
pEC369	0.697	112
pBN31-cfrD	0.633	112
pP47-61	0.658	112
pFYY063-optrA-70K	0.655	112
pR05720-3	0.738	112
pEFS108_1	0.611	112
p3-38	2.217	96

## 4 Discussion

The dissemination of ARGs or virulence factors through conjugative T4SSs across bacterial phylogenetic barriers is a global concern. The expression of T4SS proteins, the T4SS assembly, and DNA transfer require considerable energy, which emphasizes the need to regulate this energy-consuming process. The Inc18 group of plasmids, in this study represented by the prototype conjugative plasmid pIP501, contains multiple resistance plasmids with a broad host range among Gram-positive bacteria. Its repressor TraN has been proposed to be involved in regulation of the expression of the *tra* operon encoding the conjugative T4SS (Goessweiner-Mohr et al., 2014a; Kohler et al., 2018a).

Here, we validated the role of the putative TraN key residues R23, N24, Q28, G47, G48, H82, R86, G100, and K101 involved in specific TraN binding to its cognate pIP501 DNA. We performed *in vivo* biparental mating assays employing *traN* variants and *in vitro* MST measurements to decipher the sequence-specific DNA binding affinity of the TraN variants. Additionally, the study examined the putative functional role of TraN in the T4SS complex and its prevalence among plasmids from Gram-positive bacteria.

All nine residues were substituted by alanine to investigate the role of putative TraN key residues involved in binding to a specific DNA sequence (oBS) upstream of *oriT*_pIP501_ (Kohler et al., 2018a). For complementation studies, seven pEU327-RBS-*traN* variants with single (Q28A, H82A, and K101A) or multiple alanine substitutions (N23A-R24A-Q28A, G47A-G48A, H82A-R86A, and G100A-K101A) were generated. The pIP501∆*traN* complementation with TraN_R23A-N24A-Q28A, TraN_H82A-R86A, and TraN-G100A-K101A led to a significant increase of transfer by more than 1.8 log compared to the single amino acid substitutions, with the TraN_G100A-K101A variant exhibiting the highest transfer rate. The TraN double-substituted variant TraN_G47A-G48A only resulted in an increase of the transfer rate by less than one order of magnitude compared to original pIP501. The Gly47 and Gly48 only exhibit one direct protein-DNA interaction, which is potentially not affected by the Gly to Ala substitution. However, the alanine side chain might increase the sterical hindrance at the protein-DNA interface, leading to slightly weaker binding. We propose that the binding strength and specificity of TraN is mainly mediated by the residues forming direct base contacts in the major groove (Arg23, Asn24, Gln28, His82, and Arg86) or in the central minor groove (Lys101) of the dsDNA.

As the multiple-substituted TraN variants had the highest effect on pIP501 transfer frequency, MST was used to analyze their binding affinity to the specific TraN binding site (oBS) of the P_*tra*_ promoter. In general, the data from the *in vitro* DNA binding assays correlated well with the results from the mating assays, showing that TraN exhibited the lowest K_d_ value reflecting the strongest binding and thus proper regulation. The K_d_ of TraN bound to oBS DNA was similar to the K_d_ obtained with ITC measurements (Goessweiner-Mohr et al., 2014a). We found that residues H82, R86, G100, and K101 are most important for mediating high affinity binding of TraN to its oBS. The strong impact of the substitutions in the TraN_G100A-K101A variant on K_d_ is most likely due to the substitution of the Lys101 residue, which reaches deep into the minor groove of the dsDNA and forms a network of direct and indirect base contacts. The Gly100 residue enables the formation of a tight β-turn and is apparently necessary for the correct placement of Lys101 in the minor groove, as the effect of the double mutation is much more prominent than of the single mutation of TraN_K101A.

The His82 and Arg86 residues reach into the major groove of the oBS DNA and form an intricate network of direct and indirect base contacts. We observed similar DNA binding affinities for the TraN_H82A-R86A and the TraN_G100A-K101A variant. The key residues of the N-terminal recognition helix (motif 1), Arg23, Asn24, and Gln28 are positioned in the major groove of the second oBS half site and form an extended network of base interactions (Kohler et al., 2018a). However, the substitutions in the triple-substituted variant TraN_R23A-N24A-Q28A seem to affect the binding affinity less than the H82A-R86A double-substituted variant of the C-terminal recognition helix (motif 2). These differences in binding affinities between the N- and the C-terminal domains of TraN agree with previous observations that the C-terminal recognition helix (motif 2) contributes more to the thermal stability of the TraN-oBS complex than motif 1. In addition, calculations based on the complex structure yielded a higher free energy of assembly for the C-terminal domain than the N-terminal domain (Kohler et al., 2018a). The TraN_G47A-G48A variant showed only a small decrease in binding affinity compared to TraN. The crystal structure of TraN-oBS complex revealed a tight β-turn comprising residues G48 and G47, where the amide group of G48 forms a hydrogen bond to a phosphate group of the DNA backbone and the carboxyl group forms a hydrogen bond to Lys101, thus stabilizing the conformation of this minor groove binder. The substitutions of glycine by alanine do not compromise the hydrogen bonding pattern but may lead to a slight steric hindrance upon DNA binding, thus explaining the observed effects on binding affinity and transfer rates.

Studies on the expression levels of TraN variants compared to TraN showed that all variants are expressed to almost the same extent, except for TraN_H82A and TraN_H82A-R86A (Supplementary Figure S2), which also showed the lowest affinity to the anti-TraN antibody (Supplementary Figure S1C). Considering that pEU327 is an overexpression plasmid in contrast to pIP501, we are confident that the differences in transfer rate originate from the introduced mutation(s). This is supported by the results of the MST measurements, where the changes in binding affinity correlated well with the results of the mating assays. CD measurements showed similar spectra for all TraN variants and the native TraN, suggesting that the secondary structure content and the overall fold remain largely unchanged among all variants.

The pIP501-encoded T4SS consists of 15 Tra proteins, of which eight (TraB, TraC, TraF, TraH, TraI, TraK, TraL, and TraM) are likely involved in the buildup of the MPF complex, which serves as an envelope-spanning translocation channel. We investigated whether TraN can interact with other pIP501 T4SS components, as yeast two-hybrid studies had demonstrated interactions of TraN with several Tra proteins (Abajy et al., 2007). There, TraN has been reported to interact with itself, the ATPase TraE, the lytic transglycosylase TraG, and the membrane-associated VirB8-like TraH (Abajy et al., 2007). The pull-down assays in our study demonstrated coelution of TraE, TraF, TraG, TraH, TraJ, TraK, TraM, and TraO with TraN. Thus, they confirmed all these previously identified interactions. However, it should be mentioned that, for all tested proteins except TraN itself, the amount of protein in the elution fraction was significantly lower than in the flow-through fraction. We want to point out that not all Tra proteins pulled down necessarily interact directly with TraN, particularly those forming part of the MPF complex. Considering the exclusive presence of TraN in the cytosolic fraction (Goessweiner-Mohr et al., 2014a), we propose that TraN is not part of the MPF complex but rather forms direct or indirect interactions with components of the T4SS complex. This might clarify why certain putative MPF-forming Tra proteins, such as TraF, TraK, and TraM, were coeluted. In the case of TraM, coelution could occur since both proteins exhibit non-sequence-specific DNA binding properties, as recently demonstrated for the N-terminal domain of TraM (T. Berger and W. Keller, unpublished data). These results are corroborated by the high-resolution mass spectrometry analysis of the pull-down fractions, yielding significant signals for the TraN coelution of TraE, TraF, TraH, TraJ, TraK, and TraM.

*In silico* searches using tBLASTn identified plasmid-encoded TraN homologs exclusively in Firmicutes, with 81% of these plasmids containing an *oriT*. None of the putative TraN key amino acids which have been investigated in this study are affected in the TraN homologs, thus implying an important role for these residues.

The tBLASTn-based alignments using pIP501 T4SS proteins as query confirmed the presence of TraN homologs within putative T4SS gene clusters for over 65% of the selected 54 plasmids. Furthermore, plasmids were not exclusively found to belong to the Inc18 group but also to RepA_N and Rep_3 replicon families, underlining the widespread presence of pIP501-like T4SS. Most TraN homologs were found on multiple resistance Inc18 plasmids known to disseminate to a wide range of Gram-positive hosts (Kohler et al., 2018b; Nwaiwu, 2022; Zhu et al., 2022). Inc18 plasmids have been found in diverse Firmicutes isolated from food, healthcare facilities, livestock manure, or the environment (Biggel et al., 2021; Parra-Flores et al., 2021; Jozefíková et al., 2022; Tang et al., 2023). Three putative conjugative plasmids (plas1, pL15-b, and pW208) encoding a TraN homolog were affiliated to the RepA_N family. These multi-resistance plasmids are frequently found in staphylococci, enterococci, or streptococci (Weaver et al., 2009; Zhang et al., 2022). pL9 from the Rep_3 plasmid family also encodes a TraN homolog. These plasmids are frequently found in *E. faecalis* and *E. faecium* as well as in streptococci and staphylococci (Jensen et al., 2010; Mikalsen et al., 2015).

Structurally, the N-terminal domain of TraN shares similarities with the Xis excisionase from Tn*916*, and with a putative excisionase from *Klebsiella pneumoniae* (Goessweiner-Mohr et al., 2014a; Grohmann et al., 2016). Additionally, we identified a structurally similar putative DNA binding protein, BldC, from streptomycetes, which is related to MerR regulators (Savitzky and Golay, 1964; Schumacher et al., 2018) with an overall protein sequence identity of 18.3%. BldC (PDB: 6AMA) preferentially aligns with the C-terminal half of TraN (Supplementary Figure S3).

**FIGURE 8:**
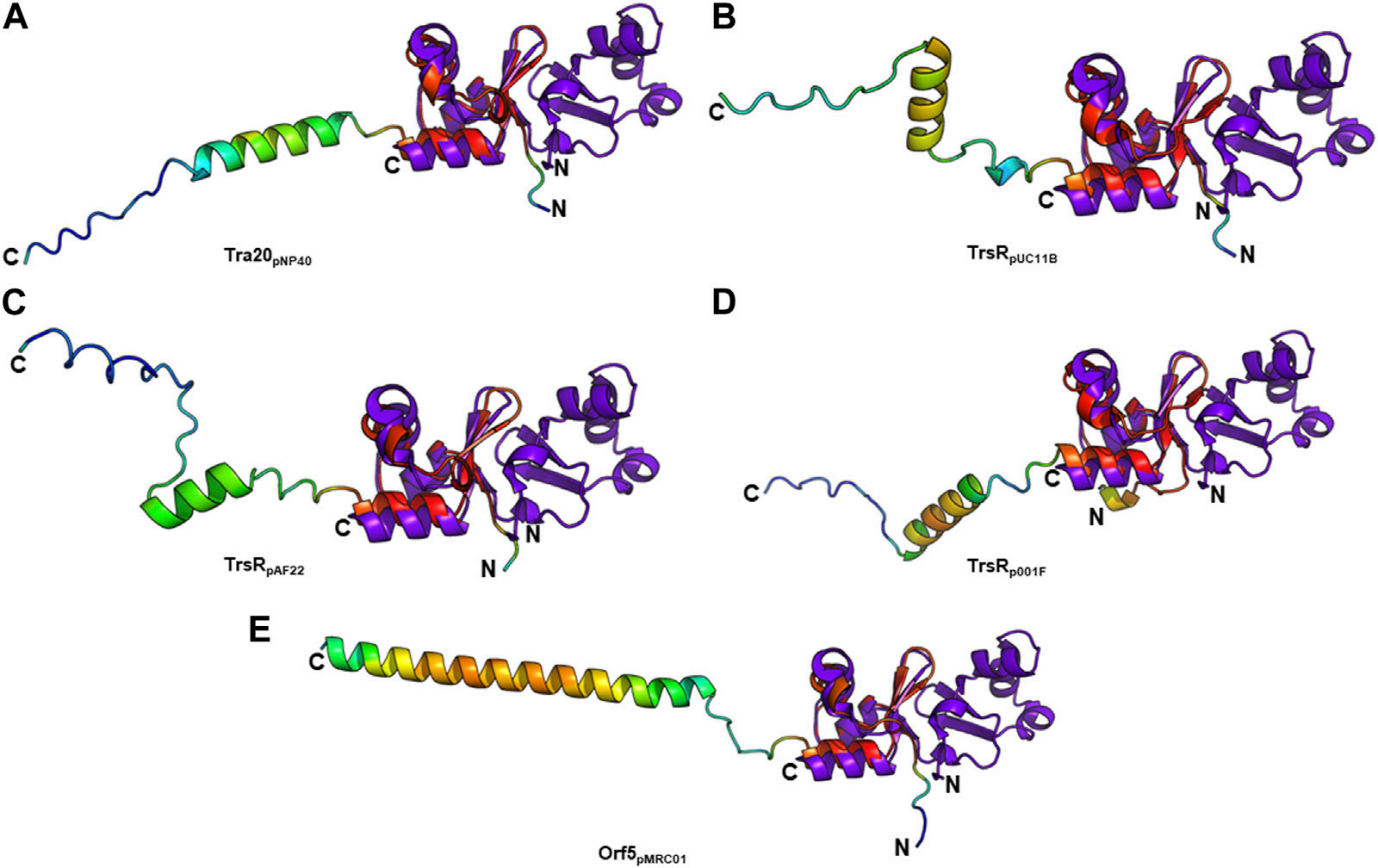
Structural alignment of TraN_pIP501_ (PDB: 4P0Z) with **(A)** Tra20_pNP40_, **(B)** TrsR_pUC11B_, **(C)** TrsR_pAF22_, **(D)** TrsR_p001F_, and **(E)** Orf5_pMRC01_. TrsR_pUC08B_ is 100% identical to TrsR_pUC11B_, and TrsR_pIBB477a_ to Orf5_pMRC01_. **(A**–**E)** Homologs are shown in rainbow, colored by pLDDT (per-residue local distance difference test) score (red = high pLDDT = high confidence; blue = low pLDDT = low confidence), TraN_pIP501_ in purple blue. All aligned proteins resemble the C-terminal half of TraN_pIP501_.

T4SS transcriptional regulators have been recently discovered in lactococcal plasmids which exhibit similarities to the MerR family of transcriptional regulators. Conjugative transfer of some of these plasmids—pNP40 (Harrington and Hill, 1991; O’Driscoll et al., 2006), pMRC01 (Coakley et al., 1997), pAF22 (Fallico et al., 2012), and pUC11B (Ortiz Charneco et al., 2023)—has been experimentally confirmed. Other lactococcal plasmids such as pUC08B, pIBB477a, and p001F have been recently discovered and categorized as pMRC01/pAF22-like conjugative plasmids (Ortiz Charneco et al., 2023). PlasmidFinder assigned pMRC01, pAF22, pUC08B, pUC11B, and pIBB477a to the Rep_3 replicon family and pNP40 to the RepA_N family. TraR and Tra20 from the conjugative *Lactococcus lactis* plasmid pNP40 were postulated as transcriptional repressors of the conjugative cluster (Ortiz Charneco et al., 2021). Both proteins exhibit low amino acid sequence identity to TraN (∼26% and ∼20%, respectively). However, structure prediction only detected a structural similarity with Tra20_pNP40_ (Figure 8A). TrsR was proposed as repressor of the *tra* operon of the conjugative pMRC01/pAF22-like pUC11B plasmid (Ortiz Charneco et al., 2023). A *trsR* ^*-*^ mutant led to a significant increase in pUC11B transfer in biparental mating assays. TrsR_pUC11B_ only has low similarity with TraN on amino acid level (∼22%) but displays structural similarities to the C-terminal half of TraN (Figure 8B). In contrast to TraN, the predicted structures of these proteins show an additional elongated polypeptide stretch with low α-helix propensity (Figure 8). It is worth mentioning that the MPF channel protein TrsB_pUC11B_ has been postulated as homolog of TraB (∼57% identity on amino acid level) (Berger et al., 2022). As TrsR_pUC08B_ is 100% identical to TrsR_pUC11B_, only one protein is depicted here.

The TrsR_pUC11B_-like homologs of pAF22 (WP_015062887.1) and p001F (WP_095348652.1) from *Lactococcus* share ∼22% and ∼32% amino acid identity with TraN_pIP501_, respectively. As depicted in Figures 8C,D, both proteins also show high similarity to the C-terminal half of the TraN structure.

The *tra* region of pMRC01 is similar to that of the Inc18 plasmids pIP501 and pRE25, and the staphylococcal plasmids pSK41 and pG01 (Grohmann et al., 2003). A gene located downstream of the relaxase gene in the *tra* region of pMRC01 is also similar to members of the MerR family of transcriptional regulators (Ortiz Charneco et al., 2023). The respective Orf5 protein also structurally resembles the C-terminal TraN_pIP501_ half (Figure 8E). pIBB477a encodes a gene 100% identical to *orf5*_pMRC01_. The conjugative gene cluster of pIBB477a displays a notable level of sequence identity with pMRC01 (>85% amino acid identity) (Ortiz Charneco et al., 2023). All five predicted structures (Tra20_pNP40_, TrsR_pUC11B_, TrsR_pAF22_, TrsR_p001F_, and Orf5_pMRC01_) exhibit the same wHTH fold as the C-terminal domain of TraN consisting of three antiparallel β-strands as well as the helix-turn-helix motif with an additional C-terminal α-helix (Figure 8). Thus, structural features of the TraN fold are not only found in excisionases (Goessweiner-Mohr et al., 2014a) but also in plasmid-encoded proteins likely involved in the transcriptional regulation of conjugative transfer.

In conclusion, this study demonstrated the importance of distinct TraN amino acid residues responsible for effective sequence-specific binding of pIP501 DNA, thereby regulating *tra* operon expression and conjugative transfer. Based on the mating assays, residues Arg23, Asn24, Gln28, His82, Arg86, Gly100, and Lys101 contribute to the regulatory function of TraN, whereas Gly47 and Gly48 appear to play a minor role and likely contribute to binding strength rather than specificity.

## Data Availability

The original contributions presented in the study are included in the article/Supplementary Material; further inquiries can be directed to the corresponding authors.
