# Supplementary material for: Effect of TraN key residues involved in DNA binding on pIP501 transfer rates in Enterococcus faecalis
Source: Front Mol Biosci. 2024 Feb 6;11:1268647. doi: 10.3389/fmolb.2024.1268647 (PMC10877727; doi:10.3389/fmolb.2024.1268647)
Supplement: Supplementary file 1 [file DataSheet1.pdf]

Supplementary Material

Supplementary Table S1 Bacterial strains and plasmids used in this work.

strain or plasmid	genotype or description	selection	reference or source
**strains**			
Priestia megaterium			
MS941	Δ*nprM*	-	Mobitec GmbH, Göttingen, Germany
Escherichia coli			
DH5α	*fhuA2* Δ*(argF-lacZ) U169 phoA glnV44* Φ*80*Δ *(lacZ)M15 gyrA96 recA1 relA1 endA1 thi-1 hsdR17*	-	New England Biolabs, Frankfurt am Main, Germany
Lemo21 (DE3)	*fhuA2 [lon] ompT gal (λ DE3) [dcm]* Δ*hsdS/* pLemo (Cam^R) *λ DE3 = λ sBamHIo* Δ*EcoRI-B int::(lacI::PlacUV5::T7 gene1) i21* Δ*nin5* pLemo = pACYC184-*PrhaBAD-lysY*	-	New England Biolabs, Frankfurt am Main, Germany
Enterococcus faecalis			
JH2-2	Derivative of *E. faecalis* JH2, Rif^R, Fus^R	Fus (50 µg/mL)	(Jacob and Hobbs, 1974)
OG1X	Protease-negative mutant of *E. faecalis* OG1-10, Sm^R	Sm (1.5 mg/mL)	(Ike et al., 1983)
**plasmids**			
pEU327	*E. coli/*G+ bacteria shuttle plasmid, Spec^R, *xylA* promoter	Spec (100 µg/mL)* Spec (500 µg/mL)**	(Eichenbaum et al., 1998)
pEU327-RBS-*traN*	pEU327 with RBS-*traN*, Spec^R	Spec (100 µg/mL)* Spec (500 µg/mL)**	This study
pEU327-RBS-*traN*_Q28A	pEU327 with RBS-*traN*_Q28A, Spec^R	Spec (100 µg/mL)* Spec (500 µg/mL)**	This study
pEU327-RBS-*traN*_H82A	pEU327 with RBS-*traN*_H82A, Spec^R	Spec (100 µg/mL)* Spec (500 µg/mL)**	This study
pEU327-RBS-*traN*_K101A	pEU327 with RBS-*traN*_K101A, Spec^R	Spec (100 µg/mL)* Spec (500 µg/mL)**	This study
pEU327-RBS-*traN*_R23A-N24A-Q28A	pEU327 with RBS-*traN*_R23A-N24A-Q28A, Spec^R	Spec (100 µg/mL)* Spec (500 µg/mL)**	This study
pEU327-RBS-*traN*_G47A-G48A	pEU327 with RBS-*traN*_G47A-G48A, Spec^R	Spec (100 µg/mL)* Spec (500 µg/mL)**	This study
pEU327-RBS-*traN*_H82A-R86A	pEU327 with RBS-*traN*_H82A-R86A, Spec^R	Spec (100 µg/mL)* Spec (500 µg/mL)**	This study
pEU327-RBS-*traN*_G100A-K101A	pEU327 with RBS-*traN*_G100A-K101A, Spec^R	Spec (100 µg/mL)* Spec (500 µg/mL)**	This study

pMGBm19	*P. megaterium*/*E. coli* shuttle vector, Cm^R, Amp^R, xylose-inducible promoter, *xyl* repressor, pBM100 replicon, ColE1 origin	Cm (35 µg/mL)	Mobitec GmbH, Göttingen, Germany
pMGBm19-RBS-*traB*-*traO*	pMGBm19 with *traB-traO*$_{pIP501}$ inserted at BamHI and SacI sites with additional 5’ RBS (AAAGGGGGAAA), Cm^R, Amp^R	Cm (35 µg/mL)	(Berger et al., 2022)
pIP501	*tra*$^+$, Cm^R, MLS^R	Cm (20 µg/mL)	(Evans and Macrina, 1983)
pIP501Δ*traN*	pIP501 *traN* in-frame deletion, Cm^R, MLS^R	Cm (20 µg/mL)	(Kohler et al., 2018)
pQTEV	P_{t4} *lacI*^{q} His_7 Amp^R	Amp (100 µg/mL)	(Scheich et al., 2004)
pQTEV-*traN*	pQTEV with *traN*, Amp^R	Amp (100 µg/mL)	This study
pQTEV-*traN*_R23A-N24A-Q28A	pQTEV with *traN*_R23A-N24A-Q28A, Amp^R	Amp (100 µg/mL)	This study
pQTEV-*traN*_G47A-G48A	pQTEV with *traN*_G47A-G48A, Amp^R	Amp (100 µg/mL)	This study
pQTEV-*traN*_H82A-R86A	pQTEV with *traN*_H82A-R86A, Amp^R	Amp (100 µg/mL)	This study
pQTEV-*traN*_G100A-K101A	pQTEV with *traN*_G100A-K101A, Amp^R	Amp (100 µg/mL)	This study
pRBBm59	*P. megaterium*/*E. coli* shuttle vector, Tet^R, Amp^R, sucrose-inducible promoter P_{sacB}, *repU* replicon, pBR322 origin	Tet (10 µg/mL)*** Amp (100 µg/mL)*	Addgene, Cambridge, Massachusetts, USA
pRBBm59-RBS-*traN*-Strep	pRBBm59 with C-terminally Strep-tagged *traN*$_{pIP501}$ and artificial RBS (AAAGGGGGAAA), Tet^R, Amp^R	Tet (10 µg/mL)*** Amp (100 µg/mL)*	This study
pRBBm59-RBS-*gfp*-Strep	pRBBm59 with C-terminally Strep-tagged *gfp*$_{pRBBm59}$ and artificial RBS (AAAGGGGGAAA), Tet^R, Amp^R	Tet (10 µg/mL)*** Amp (100 µg/mL)*	This study

Amp^R, ampicillin resistance; Cm^R, chloramphenicol resistance; Em^R, erythromycin resistance; Fus^R, fusidic acid resistance; $Gent^R$, gentamicin resistance; Kan^R, kanamycin resistance; MLS^R, macrolide-lincosamide-streptogramin B resistance, Rif^R, rifampicin resistance; $Spec^R$, spectinomycin resistance; Sm^R, streptomycin resistance; Tet^R, tetracycline resistance; RBS, ribosomal binding site; *tra*$^+$, transfer proficient; antibiotic concentration used for selection in * *E. coli*, ** *E. faecalis* and *** *P. megaterium* given in brackets.

Supplementary Table S2 Oligonucleotides used in this work.

name	sequence (5'-3')	nucleotide position/reference
***traN* expression cloning**		
GA_pQTEV-traN fw	CTTTATTTTCAGGGATCCGGAAAAATTAATTTAAATC AAATTTACAC	6687-6716[a]
GA_pQTEV-traN rev	TATCAACAGTCGACCCTTATCTGACATTTTTATTCATC TTTTTTTTAAAAAC	7020-7052[a]
GA_pQTEV189-190 fw	GGGTCGACTGTTGATAGATC	190-209[a]
GA_pQTEV189-190 rev	GGATCCCTGAAAATAAAGATTC	168-189[a]
***traN* complementation**		
pEU327-BstYI_RBS traN fw	CGC**GGATCT**AATACGCTAAAAAGAGAGT	6659-6677[b]
pEU327_SalI RBS traN rev	CGC**GTCGAC**TTATCTGACATTTTTATTCATC	7034-7055[b]
Mut_Ala28_V2 fw	ATACCTATCCGCAGCTTACCGTAAC	6759-6782[b]
Mut_Ala28_V2 rev	GCTGCATTCTTACCTATCCTTTCACTCAT	6729-6752[b]
Mut_traN Ala82 fw	ATACTTTGCCGCCATTTATAAACGGTTTC	6920-6948[b]
Mut_traN Ala82 rev	TCATCATTCTTTCCTAACAA	6900-6919[b]
Mut_traN Ala101 fw	TTATACAGGTGCAACATTATTCTTAACG	6977-7004[b]
Mut_traN Ala101 rev	ATGTGATCGATTCCCTCTAG	6957-6976[b]
Mut_traN_Ala23_Ala24 fw	AGGTAAGAATGCAGCATACCTATCCCAAGCTTAC	6743-6776[b]
Mut_traN_Ala23_Ala24 rev	ATCCTTTCACTCATTTCTTTTG	6721-6742[b]
Mut_traN_Ala100 fw	CATTTATACAGCAGCAACATTATTCTTAACGAAAGAA TCGC	6974-7014[b]
Mut_traN_Ala100 rev	TGATCGATTCCCTCTAGTC	6955-6973[b]
Mut_traN_Ala86_fw	CATTTATAAAGCATTTCCTCATAGACTAGAGGGAATC G	6932-6969[b]
Mut_traN_Ala86_V2 rev	GCGGCAAAGTATTCATCATTC	6911-6929[b]
Mut_traN_Ala47_Ala48 fw	TCGAAAAATTGCAGCAACAATTATTTCTCTGATAAT CCCAATAATG	6815-6861[b]
Mut_traN_Ala47_Ala48 rev	TAATTAAAATTTTTAAGTATTTCATGTTTGTTGT	6781-6814[b]
**pEU327 sequencing**		
pEU327 fw	CTTGCCAGTCACGTTACG	(Eichenbaum et al., 1998)
pEU327 rev	GATCAGCGATATCCACTTC	(Eichenbaum et al., 1998)

**pQTEV sequencing**		
Screen pQTEV fw	AATAGGCGTATCACGAGGC	4771-4789[a]
Screen pQTEV rev	CCAGATGGAGTTCTGAGGTCATT	282-304[a]
**pRBBm59 cloning**		
GA_pRBBm59-traN fw	AGGGGGAAAGGATCCATGGAAAAATTAATTTAAATCAAATTTAC	6687-6716
GA_pRBBm59-traN rev	GTGGCTCCAAGCGCTTCTGACATTTTTATTCATCTTTTTTTTAAAAAC	7020-7052
GA_pRBBm59-GFP fw	AGGGGGAAAGGATCCATGGCTAGCAAAGGAGAAG	This study
GA_pRBBm59-GFP rev	GTGGCTCCAAGCGCTTTTGTAGAGCTCATCCATG	This study
GA_pRBBm59-RBS-Strep fw	AGCGCTTGGAGCCACCCG	This study
GA_pRBBm59-RBS-Strep rev	GGATCCTTTCCCCCTTTTTACCAGATCTTC	This study
**pRBBm59 sequencing**		
Screen_pRBBm59 fw	CGCAACGTCTGGAAATCGTG	(Berger et al., 2022)
Screen_pRBBm59 rev	GCGCATTCACAGTTCTCCGC	(Berger et al., 2022)
**oligonucleotides for microscale thermophoresis**		
original binding site fw	CGGAAATGTCAGGTTAAACATATTTACTTTTATA	(Goessweiner-Mohr et al., 2014)
original binding site rev	TATAAAAGTAAATATGTTTAACCTGACATTTCCG	(Goessweiner-Mohr et al., 2014)
random DNA fw	AGGGCGCACTTATACGCAGTAACTTCGTTACTTC	(Goessweiner-Mohr et al., 2014)
random DNA rev	GAAGTAACGAAGTTACTGCGTATAAGTGCGCCCT	(Goessweiner-Mohr et al., 2014)

restriction sites are shown in **bold**

GenBank accession numbers: [a]AY243506.1, [b] AJ505823.1

All primers without reference in the table were designed in this study.

Supplementary Table S3 Final concentration of double-stranded DNA oligos in the dilution series utilized for MST measurements.

position	c(DNA) [nM]
1	12800
2	6400
3	3200
4	1600
5	800
6	400
7	200
8	100
9	50
10	25
11	12.5
12	6.25
13	3.125
14	1.563
15	0.781
16	0

Supplementary Table S4 Transfer rates obtained in biparental mating assays.

mating A: testing single point mutations in putative TraN key residues		
**donor**	**recipient**	**transfer rate (transconjugants/recipient)***
E. faecalis (pIP501)	*E. faecalis* OG1X	$6.47 \times 10^{-6} \pm 2.14 \times 10^{-6}$
E. faecalis (pIP501Δ*traN*)		$4.71 \times 10^{-4} \pm 8.69 \times 10^{-5}$
E. faecalis (pIP501Δ*traN*, pEU327-RBS-*traN*)		$1.17 \times 10^{-5} \pm 8.57\times 10^{-6}$
E. faecalis (pIP501Δ*traN*, pEU327-RBS-traN_Q28A)		$3.17 \times 10^{-5} \pm 2.14 \times 10^{-5}$
E. faecalis (pIP501Δ*traN*, pEU327-RBS-*traN*_H82A)		$4.66 \times 10^{-5} \pm 2.55 \times 10^{-5}$
E. faecalis (pIP501Δ*traN*, pEU327-RBS-*traN*_K101A)		$1.50 \times 10^{-5} \pm 7.85 \times 10^{-6}$
**mating B: testing multiple mutations in putative TraN key residues**		
**donor**	**recipient**	**transfer rate (transconjugants/recipient)***
E. faecalis (pIP501)	*E. faecalis* OG1X	$1.72 \times 10^{-5} \pm 8.46 \times 10^{-6}$
E. faecalis (pIP501Δ*traN*)		$1.72 \times 10^{-3} \pm 6.13 \times 10^{-4}$
E. faecalis (pIP501Δ*traN*, pEU327-RBS-*traN*)		$1.06 \times 10^{-5} \pm 7.45 \times 10^{-6}$
E. faecalis (pIP501Δ*traN*, pEU327-RBS-*traN*_R23A-N24A-Q28A)		$6.98 \times 10^{-4} \pm 2.33 \times 10^{-4}$
E. faecalis (pIP501Δ*traN*, pEU327-RBS-*traN*_G47A-G48A)		$3.79 \times 10^{-5} \pm 2.81 \times 10^{-5}$
E. faecalis (pIP501Δ*traN*, pEU327-RBS-*traN*_H82A-R86A)		$7.95 \times 10^{-4} \pm 2.25 \times 10^{-4}$
E. faecalis (pIP501Δ*traN*, pEU327-RBS-*traN*_G100A-K101A)		$6.86 \times 10^{-4} \pm 3.91 \times 10^{-5}$

*mean values are given with standard deviation (±SD). Transfer rates are given as the number of transconjugants per recipient cell. n = 3. Mating A corresponds to Figure 1B, mating B to Figure 1C.

Supplementary Table S5 Alignment of predicted structures of TraN variants with TraN showing the number of aligned atoms, RMSD values and the color code of Supplementary Figure S3.

**$TraN_{pIP501}$ variant**	**RMSD values [Å]**	**# of aligned C_{α} atoms**	**color code**
TraN_R23A-N24A-Q28A	0.617	112	light pink
TraN_G47A-G48A	0.588	112	teal
TraN_H82A-R86A	0.643	112	light blue
TraN_G100A-K101A	0.627	112	slate
TraN_Q28A	0.520	112	-
TraN_H82A	0.519	112	-
TraN_K101A	0.540	112	-

Supplementary Table S6 *In silico* digest of Tra proteins showing the resulting peptides for MS analysis.

Protein IDs	Unique peptides	Sequence coverage [%]	MW [kDa]	Peptide sequences
TraB	2	18.2	11.56	LVQAGNTIK; YLPSIDDPQEK
TraD	4	15.8	22.16	EMIYWEDSSR; NLASNMETSK; NLASNMETSKK; TALIAFLSK
TraE	17	28.9	75.809	AIIGNSFTK; ALEETDPKR; ALIPYTMIDK; ELSFITSK; FVFGLSGGGK; IKVDLTQEEMR; INPFQIYSR; IVLFDPEDEQTER; LCEDFETR; LLIDDSYAVPYVITK; LLNPGEYEDIYGVSPK; MENTDFPTFSDLENR; SLGGEIINLSSMSDVR; TTYLTSDYLK; TTYLTSDYLKK; VFFGLDETEAQGISNELK; YAFSDNSTELR
TraF	14	30.2	52.755	AYELDKK; DLLFIYK; EKDIPTVK; IIDNDSENIEEDMQK; IIQSLYK; LLEMNVLEK; NFFSAELQDK; NFFSAELQDKK; NFICSFFGK; NRIEVLTK; NSFLNDVEAVESLESLR; QVDIYVK; QVTATQQSAETEITILNK; TNTYLAMQNILLK
TraH	5	31.7	21.171	EISMTTVK; NDFEINQLIQIK; SIQTELADKQEEQNTNQSESEK; SSVNENEIK; TVYNYEK
TraJ	16	32.3	63.07	EYIEELEK; IANVIVSAK; ICLGGVEETTAEYFSR; KVQLASAPIFHEK; NIFVVGGPGSFK; QVNDTTIK; SIVLIDGKPYMLR; SYYLGFR; TPQFELFGNLLK; TQSYVLPNVVNNR; TSLLAESDGLILGK; VVIQPEDSK; VYTGGTSESK; YNEEGVSELDEQFER; YNPLLYIR; YVYFEYEPSAR
TraK	6	28.7	34.695	ADEATEAFESWYK; EFFGNIPMFQK; EGSGGTSPIELQTK; ESNQSETSGEATENSSQAVQGSSDHLLK; GIEPSEGIK; SFSNGDVILEINK
TraM	19	57.1	37.484	ESELLEEQIER; ESLSESITK; ESLSESITKK; ETDVINLLLNR; IILPEEENQFLNR; IILPEEENQFLNRK; IMSVTLNTK; ITFEVEPTK; KESLSESITK; NGFLVTTQPK; NQENYQSSLR; QEIPQTEIPTETVNEPSVIKK; SELSEVTHYLQQK; SVNYYGSEQTK; SVNYYGSEQTKK; VDISDWK; VKETDISQSK; VTDQHVLVYLNEPTEPTLNTQELNR; YFLTYYFSQEK
TraN	23	90.2	14.37	EASQLLGK; EASQLLGKNDEYFAHIYK; EASQLLGKNDEYFAHIYKR; EMSERIGK; EMSERIGKNR; ESLEVFK; ESLEVFKK; ESLEVFKKK; FPHRLEGIDHIYTGK; GKINLNQIYTAK; GKINLNQIYTAKEMSER; IGGTIIFSDNPNNDLSQLITAK; INLNQIYTAK; INLNQIYTAKEMSER; KIGGTIIFSDNPNNDLSQLITAK;

				LEGIDHIYTGK; LEGIDHIYTGKTLFLTK; NDEYFAHIYK; NDEYFAHIYKR; NNKHEILK; NRNYLSQAYR; NYLSQAYR; RFPHRLEGIDHIYTGK

Supplementary Table S7 Results of the MS analysis of the pull-down assays showing intensities and LFQ intensities of the detected Tra proteins.

Protein IDs	Intensity Run1	Intensity Run2	Intensity Run3	LFQ intensity Run1	LFQ intensity Run2	LFQ intensity Run3
TraB	95135	161560	74197	0	87416	0
TraD	32896	0	29145	0	0	0
TraE	354980	391820	344910	77362	85463	76151
TraF	191220	279770	180100	58381	61914	65084
TraH	74691	77760	108190	39039	51524	45572
TraJ	336860	364130	337570	66202	65571	65094
TraK	269480	292650	261170	124460	135510	121330
TraM	566880	651060	600470	138370	119310	114240
TraN	246550000	187520000	226040000	28966000	24143000	27089000

LFQ: Lable free quantification

Supplementary Table S8 Putative $TraN_{pIP501}$ homologs encoded on plasmids or transposons.

species	plasmid /transposon	accession number	plasmid replicon (Rep) family	Rep identity [%]	protein length (aa)	query coverage [%]	amino acid identity [%]	gene product and/or accession number
Enterococcus faecalis	pIP501	AJ505823.1	Inc18[a]	100	122	-	-	$TraN_{pIP501}$ CAD44394.1
Enterococcus faecalis	pV386	MZ603802.1	Inc18[a]	100	122	100	100	TraN UJJ80275.1
Enterococcus faecalis	pAMβ1	AF007787.1	Inc18[a]	100	122	100	100	OrfB AAC38599.1
Enterococcus faecalis	pRE25	X92945.2	Inc18	100	122	100	100	CAC29193.1
Enterococcus faecalis	p2[#]	CP041740.1	Inc18[a] RepA_N[b]	100 100	122	100	100	not annotated[1]
Enterococcus faecalis	pWZ1668	GQ484956.1	Inc18[a]	100	122	100	100	not annotated[2]
Enterococcus faecalis	pWZ7140	GQ484955.1	Inc18[a]	100	122	100	100	not annotated[3]
Enterococcus faecalis	pWZ909	CP041740.1	Inc18[a]	100	122	100	100	ADM24819.1
Enterococcus faecalis	pSF3	CP060803.1	Inc18[a]	100	122	100	100	QNP36475.1
Enterococcus faecalis	pBE43_3	CP110044.1	Inc18[a]	98.56	122	100	100	UYY26443.1
Enterococcus faecalis	pBE15_3	CP110066.1	Inc18[a]	98.46	122	100	100	UYY43036.1
Enterococcus faecalis	pKUB3007-3	AP018546.1	Inc18[a]	100	122	100	100	BBD29588.1
Enterococcus faecalis	pKUB3006-3	AP018541.1	Inc18[a]	100	122	100	100	BBD26547.1
Enterococcus faecalis	pS39-4-b	CP088202.1	not characterized	-	122	100	100	UQR10319.1
Enterococcus faecalis	pK190-1-B	CP116573.1	Inc18[a]	99.8	122	100	100	WCG31825.1
Enterococcus faecalis	pNBZ135-cfr-102K[##]	CP117513.1	Inc18[a]	99.27	122	100	100	WDA22678.1
Enterococcus faecium	pAVS02435_2	CP072896.1	Inc18[a]	99.93	122	100	100	QXJ66669.1
Enterococcus faecium	pELF_mdr	AP026773.1	Inc18[a]	100	122	100	100	BDR32126.1
Enterococcus faecium	p116-2	CP047329.1	Inc18[a]	99.93	122	100	100	QUR72457.1
Enterococcus faecium	pPPM1000*	AY351675.1	not characterized	-	122	100	100	AAR10432.1
Enterococcus gallinarum	pEGM181-1*	CP050484.1	Inc18[a]	99.89	122	100	100	QOG25797.1
Enterococcus hirae	pF104_1*	CP072892.1	Inc18[a]	96.17	122	100	100	QXJ64246.1

Streptococcus pyogenes	pMD101*	X66468.1	Inc18[a]	100	122	100	100	ORFθ CAA47096.1
Streptococcus pyogenes	pSM19035*	AY357120.1	Inc18[a]	100	122	100	100	AAR27210.1
Vagococcus fluvialis	p36B2_p1	CP081462.1	not characterized	-	122	100	100	UDM75526.1
Lactococcus garvieae	pkh2101	ON321837.1	Inc18[a]	98.32	122	100	100	URQ58530.1
Bacillus subtilis	pGMrib[#]	CP045673.1	Inc18[a]	99.93	122	100	100	not annotated[4]
Enterococcus faecalis	p4	MH830362.1	Inc18[a] RepA_N[b]	99.2 97.05	122	100	99.18	QFX76044.1
Enterococcus faecalis	pW208*	CP096049.1	RepA_N[b]	97.05	122	100	99.18	UPQ25550.1
Enterococcus faecalis	pL9	CP041776.2	Rep_3[c] Inc18[a]	98.52 97.46	122	100	99.18	QTO65521.1
Enterococcus faecalis	pEFS36_2	CP085293.1	Inc18[a]	100	122	100	99.18	UDM45511.1
Enterococcus faecalis	pDM86-poxtA	CP116965.1	Inc18[a]	100	122	100	99.18	WCO77992.1
Enterococcus faecalis	pAT39-b*	CP097040.1	Inc18[a]	100	122	100	99.18	UQF47389.1
Enterococcus faecium	pEF12-0805	KY579372.1	Inc18[a]	100	122	100	99.18	ARQ19301.1
Enterococcus faecium	pAT02-c	CP097064.1	Inc18[a]	95.78	122	100	99.18	UQF70175.1
Streptococcus suis	pStrcfr*	KC844836.1	not characterized	-	122	100	99.18	AGO02194.1
Staphylococcus aureus	Tn*6349*	MH746818.1	not characterized	-	122	100	99.18	TraN QBG78974.1
Enterococcus faecium	pE35048-oc	MF580438.1	Inc18[a]	99.2	123	100	98.37	AVH81513.1
Enterococcus faecium	pA10290_P2	CP059757.1	Inc18[a]	77.65	122	100	98.36	QMX45230.1
Vagococcus lutrae	pBN31-cfrD	CP081834.1	Inc18[a]	98.32	122	100	94.26	QZN89724.1
Enterococcus casseliflavus	pEC369[##]	CP032740.1	Inc18[a]	98.07	122	100	87.7	AYJ46916.1
Enterococcus faecalis	pAT40b-a	CP097035.1	Inc18[a]	98.2	117	95	96.55	UQF32112.1
Enterococcus faecalis	pL15-B[##]	CP072514.1	RepA_N[b]	97.05	117	95	96.55	QTO36079.1
Enterococcus faecium	pVEF3[#]	AM931300.1	Inc18[a]	100	117	95	96.55	CAP62658.1
Enterococcus faecium	pP47-61	CP091102.1	Inc18[a]	96.37	117	95	96.55	UJM32022.1
Enterococcus faecium	pT17-1-optrA-57k	CP109840.1	Inc18[a]	96.37	117	95	96.55	UYV50164.1
Enterococcus faecium	pK80-15-b-B	CP116551.1	Inc18[a]	77.3	117	95	96.55	WCG67835.1
Enterococcus faecium	pW6-2	CP118549.1	Inc18[a]	96.2	117	95	96.55	WDW18949.1
Enterococcus faecium	pR05720-3	CP064409.1	not characterized	-	117	95	91.38	QUM65744.1

Enterococcus lactis	pY3-1	CP084247.1	Inc18[a]	99.92<br>96.89	117	95	96.55	UBX35715.1
Enterococcus casseliflavus	pFYY063-optrA-70K	CP116030.1	Inc18[a]	98.53	117	95	96.55	WBY93795.1
Enterococcus gilvus	pCR1b	CP072514.1	Inc18[a]	98.67	117	95	96.55	AXG40760.1
Lactococcus lactis subsp. *lactis*	plas1[#]	CP064340.1	RepA_N[b]<br>Inc18[a]	99.62<br>96.97	117	95	96.55	not annotated[5]
Enterococcus faecalis	pEFS108_1[##]	CP085295.1	Inc18[a]	100	127	93	98.25	UDM48174.1
Enterococcus thailandicus	pW3[##]	JQ911739.1	not characterized	-	99	79	100	AFW17870.1
Enterococcus thailandicus	p3-38[##]	JQ911740.1	not characterized	-	99	79	97.06	AFW17890.1

TraN homologs were detected via a tBLASTn search using the NCBI database. Search criteria are described in detail in 2.12 in the main text. Percentage amino acid identities were calculated with the BLAST algorithm. [1] sequence position (CP041740.1) nucleotides 254-662, [2] sequence position (GQ484956.1) 1,067-1,435, [3] sequence position (GQ484955.1) 1,067-1,435, [4] sequence position (CP045673.1) 3,524-3,889, [5] sequence position (CP064340.1) 215,281-215,634. Replicon families are presented by respective replication (*rep*) genes: [a] Inc18 family (*rep1*, *rep2*, *repUS1*), [b] RepA_N family (*repUS15*, *rep8b*, *rep9b*), [c] Rep_3 family (*repUS35*) and [d] for Rep_trans family (*rep7a*). Unlabeled plasmids contain a predicted *oriT* and a complete pIP501-like T4SS (TraA – TraO). * denotes plasmids that do not contain an *oriT*, as determined with oriTfinder (https://bioinfo-mml.sjtu.edu.cn/oriTfinder/), [#] denotes plasmids that do not encode a pIP501-like T4SS (TraA – TraO) or missing at least one pIP501-like signature protein such as the relaxase TraA, the ATPase TraE or the coupling protein TraJ, [##] denotes plasmids encoding for 14 out of 15 pIP501-like T4SS proteins (TraA – TraN) lacking a TraO-like protein.

Supplementary Table S9 $TraE_{pIP501}$ and $TraJ_{pIP501}$ homologs in putative non-conjugative plasmids encoding *traN*-like sequences. All plasmids lack a TraA-like relaxase and four out of five plasmids an *oriT*-like sequence.

plasmid	homologs to $T4SS_{pIP501}$	protein identity [%]	*oriT* predicted by oriTfinder*
pIP501[1,2]	TraA<br>TraE<br>TraJ	100 ($TraA_{1-661}$)<br>100 ($TraE_{1-653}$)<br>100 ($TraJ_{1-551}$)	yes
pSM19035[3]	no TraA<br>no TraE<br>hypothetical protein[a]	-<br>-<br>99 ($TraJ_{1-551}$)	no
pF104_1[4]	no TraA<br>no TraE<br>T4SS conjugative DNA transfer family protein	-<br>-<br>99 ($TraJ_{1-551}$)	no
pEGM181-1[5]	no TraA<br>TraE<br>T4SS conjugative DNA transfer family protein	-<br>99 ($TraE_{1-653}$)<br>99 ($TraJ_{1-551}$)	no
pGMrib[6]	no TraA<br>no TraE<br>not annotated[b]	-<br>-<br>99 ($TraJ_{1-551}$)	no
p2[7]	no TraA<br>no TraE<br>not annotated[c]	-<br>-<br>99 ($TraJ_{1-551}$)	yes

*oriTfinder (https://bioinfo-mml.sjtu.edu.cn/oriTfinder/) and tBLASTn algorithm from NCBI (https://blast.ncbi.nlm.nih.gov/Blast.cgi) were used to search for putative TraA, TraE, and TraJ homologs and an *oriT*. GenBank accession numbers: [1] AJ505823.1, [2] L39769.1, [3] AY357120.1, [4] CP072892.1, [5] CP050484.1, [6] CP045673.1, [7] CP041740.1, [a] AAR27217.1, [b] sequence position (CP045673.1) 17,990-19,510, [c] sequence position (CP041740.1) 3,517-4,869.

Expression and purification of TraN variants

The gels monitoring expression and purification of all TraN variants are shown in Figure 2 in the main manuscript. An alignment of His-TraN and its variants is presented in Figure S1A highlighting the mutated residues. Figure 1B depicts the chromatograms of the analytical SEC runs. In addition, the 260/280 ratio reflects DNA- free protein as it is below the threshold of 0.57. In Supplementary Figure 1C we show the blot of the His-TraN variants probed with anti-TraN antibody. Although we loaded the same amounts of protein in every lane the intensities of the bands differ, indicating that the antibody does not recognize every variant to the same extent.

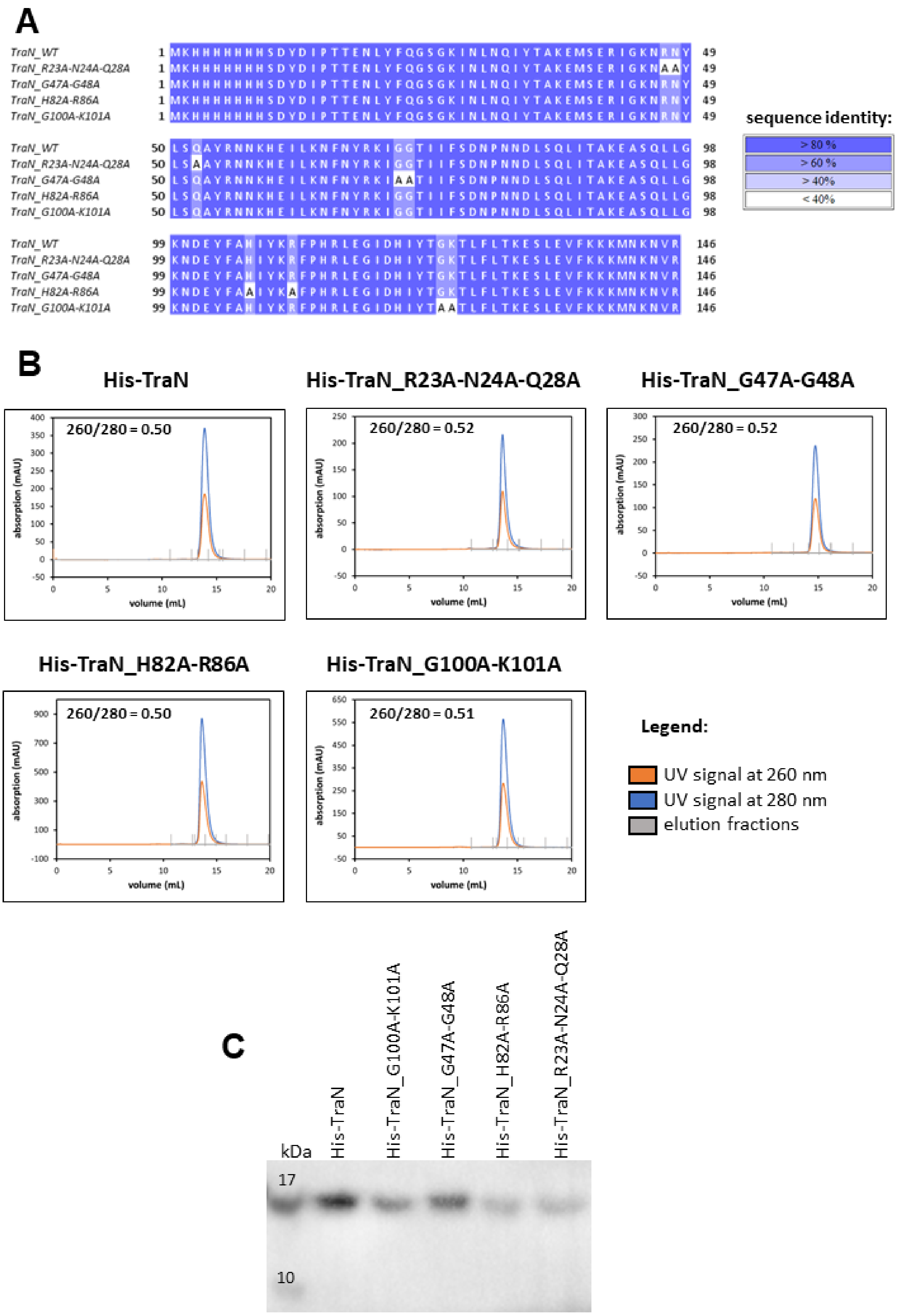
A
TraN_WT 1 MKHHHHHHHSDYDIPTTENLYFQGSGKINLNQIYTAKEMSERIGKNRNY 49
TraN_R23A-N24A-Q28A 1 MKHHHHHHHSDYDIPTTENLYFQGSGKINLNQIYTAKEMSERIGKNAAY 49
TraN_G47A-G48A 1 MKHHHHHHHSDYDIPTTENLYFQGSGKINLNQIYTAKEMSERIGKNRNY 49
TraN_H82A-R86A 1 MKHHHHHHHSDYDIPTTENLYFQGSGKINLNQIYTAKEMSERIGKNRNY 49
TraN_G100A-K101A 1 MKHHHHHHHSDYDIPTTENLYFQGSGKINLNQIYTAKEMSERIGKNRNY 49
TraN_WT 50 LSQAYRNNKHEILKNFNYRKIGGTIIFSDNPNNDLSQLITAKEASQLLG 98
TraN_R23A-N24A-Q28A 50 LSAAYRNNKHEILKNFNYRKIGGTIIFSDNPNNDLSQLITAKEASQLLG 98
TraN_G47A-G48A 50 LSQAYRNNKHEILKNFNYRKIAATIIFSDNPNNDLSQLITAKEASQLLG 98
TraN_H82A-R86A 50 LSQAYRNNKHEILKNFNYRKIGGTIIFSDNPNNDLSQLITAKEASQLLG 98
TraN_G100A-K101A 50 LSQAYRNNKHEILKNFNYRKIGGTIIFSDNPNNDLSQLITAKEASQLLG 98
TraN_WT 99 KNDEYFAHIYKRFPHRLEGIDHIYTGKTLFLTKESLEVFKKKMNKNVR 146
TraN_R23A-N24A-Q28A 99 KNDEYFAHIYKRFPHRLEGIDHIYTGKTLFLTKESLEVFKKKMNKNVR 146
TraN_G47A-G48A 99 KNDEYFAHIYKRFPHRLEGIDHIYTGKTLFLTKESLEVFKKKMNKNVR 146
TraN_H82A-R86A 99 KNDEYFAAIYKAFPHRLEGIDHIYTGKTLFLTKESLEVFKKKMNKNVR 146
TraN_G100A-K101A 99 KNDEYFAHIYKRFPHRLEGIDHIYTAATLFLTKESLEVFKKKMNKNVR 146
sequence identity:
> 80 %
> 60 %
> 40%
< 40%
B
His-TraN
260/280 = 0.50
His-TraN_R23A-N24A-Q28A
260/280 = 0.52
His-TraN_G47A-G48A
260/280 = 0.52
His-TraN_H82A-R86A
260/280 = 0.50
His-TraN_G100A-K101A
260/280 = 0.51
absorption (mAU)
volume (mL)
Legend:
UV signal at 260 nm
UV signal at 280 nm
elution fractions
C
kDa
His-TraN
His-TraN_G100A-K101A
His-TraN_G47A-G48A
His-TraN_H82A-R86A
His-TraN_R23A-N24A-Q28A
17
10

Supplementary Figure S1 Purification of TraN$_{pIP501}$ and its variants. (A) Multiple sequence alignment of TraN and its variants colored according to sequence identity. Alignment was performed with ClustalOmega (Waterhouse et al., 2009; Madeira et al., 2022). (B) Chromatograms showing the elution profile of purified His-TraN and its variants. The presence of a sharp, single peak proves the purity of the proteins. The 260/280 ratio shown in the respective chromatograms indicates that the proteins do not contain any DNA. (C) Western Blot analysis of purified His-TraN and its variants expressed in *E. coli* Lemo21 (DE3) cells. Immunoblot using a polyclonal anti-TraN antibody to check whether the alanine substitutions change the binding affinity of the anti-TraN antibody. A molecular mass standard protein ladder (Blue Prestained Protein Standard, New England Biolabs) was used for molecular weight (MW) reference. For each protein, 0.75 µg was loaded. The lanes are labeled with the respective protein. The 7×His-tag adds 2.8 kDa to the MW of TraN and its variants. Therefore, the bands appear at about 17 kDa.

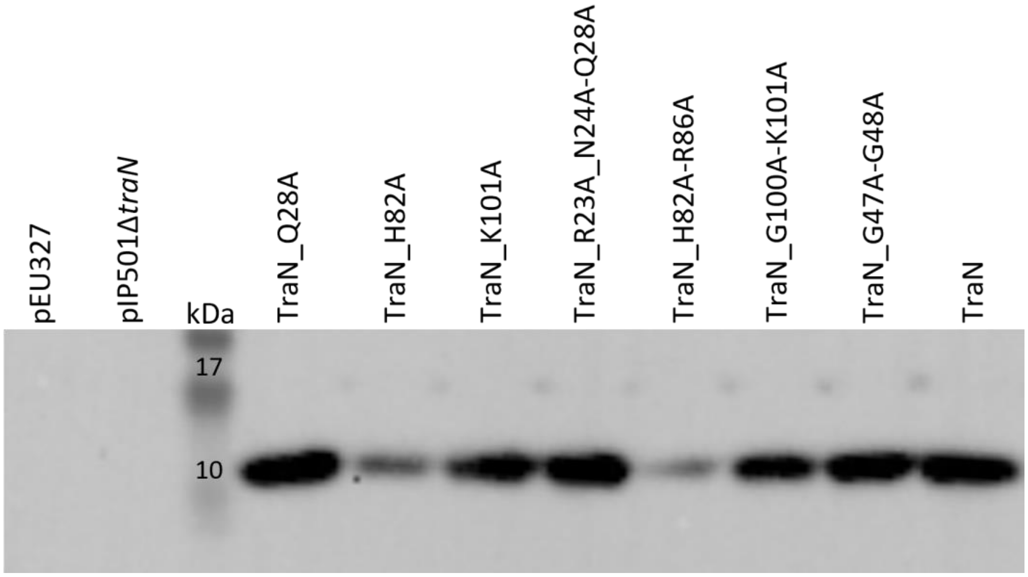

Supplementary Figure S2 Western Blot analysis of expressed TraN variants in *E. faecalis* JH2-2. Immunoblot using a polyclonal anti-TraN antibody to check expression of TraN and its variants in *E. faecalis* JH2-2. The lanes are labeled with the expressed protein. For the negative controls, the plasmid name, pEU327 (lane 1), pIP501Δ*traN* (lane 2) is given. The molecular mass standard, protein ladder (Blue Prestained Protein Standard, New England Biolabs) was used as molecular weight reference.

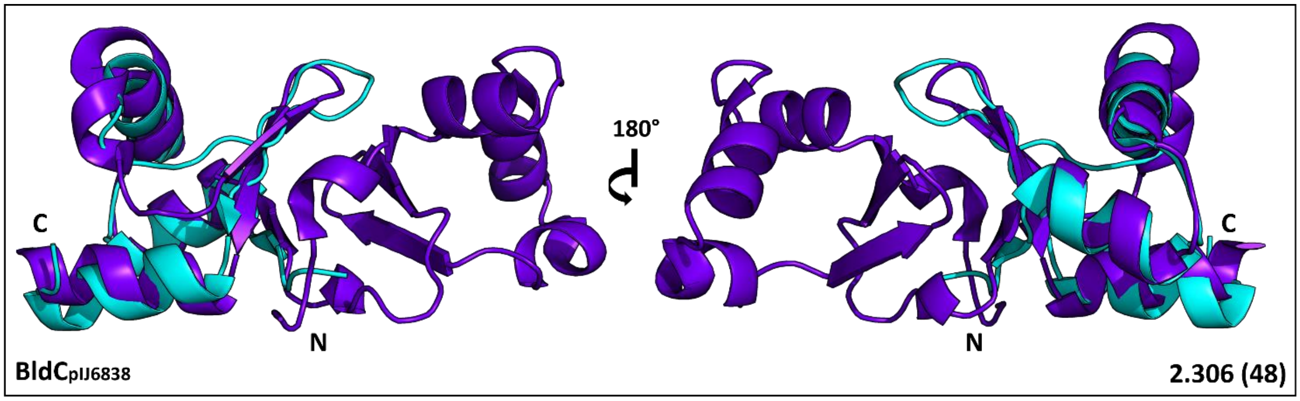

Supplementary Figure S1 Structural alignment of TraN$_{pIP501}$ (PDB: 4P0Z, purple) with BldC (PDB: 6AMA, cyan). Only one of the eleven identical DNA-bound proteins in the asymmetric unit was used for the alignment. The bottom right corner displays the RMSD value and in brackets the number of aligned C_{α} atoms between TraN$_{pIP501}$ and BldC.

REFERENCES

Berger, T. M. I., Michaelis, C., Probst, I., Sagmeister, T., Petrowitsch, L., Puchner, S., et al. (2022). Small Things Matter: The 11.6-kDa TraB Protein is Crucial for Antibiotic Resistance Transfer Among Enterococci. *Front Mol Biosci* 9, 867136. doi: 10.3389/fmolb.2022.867136

Eichenbaum, Z., Federle, M. J., Marra, D., Vos, W. M. de, Kuipers, O. P., Kleerebezem, M., et al. (1998). Use of the lactococcal *nisA* promoter to regulate gene expression in gram-positive bacteria: comparison of induction level and promoter strength. *Appl Environ Microbiol* 64, 2763–2769. doi: 10.1128/AEM.64.8.2763-2769.1998

Evans, R. P., and Macrina, F. L. (1983). Streptococcal R plasmid pIP501: endonuclease site map, resistance determinant location, and construction of novel derivatives. *J Bacteriol* 154, 1347–1355. doi: 10.1128/jb.154.3.1347-1355.1983

Goessweiner-Mohr, N., Eder, M., Hofer, G., Fercher, C., Arends, K., Birner-Gruenberger, R., et al. (2014). Structure of the double-stranded DNA-binding type IV secretion protein TraN from *Enterococcus*. *Acta Crystallogr D Biol Crystallogr* 70, 2376–2389. doi: 10.1107/S1399004714014187

Ike, Y., Craig, R. A., White, B. A., Yagi, Y., and Clewell, D. B. (1983). Modification of *Streptococcus faecalis* sex pheromones after acquisition of plasmid DNA. *Proc Natl Acad Sci U S A* 80, 5369–5373. doi: 10.1073/pnas.80.17.5369

Jacob, A. E., and Hobbs, S. J. (1974). Conjugal transfer of plasmid-borne multiple antibiotic resistance in *Streptococcus faecalis* var. *zymogenes*. *J Bacteriol* 117, 360–372. doi: 10.1128/jb.117.2.360-372.1974

Kohler, V., Goessweiner-Mohr, N., Aufschnaiter, A., Fercher, C., Probst, I., Pavkov-Keller, T., et al. (2018). TraN: A novel repressor of an *Enterococcus* conjugative type IV secretion system. *Nucleic Acids Res* 46, 9201–9219. doi: 10.1093/nar/gky671

Madeira, F., Pearce, M., Tivey, A. R. N., Basutkar, P., Lee, J., Edbali, O., et al. (2022). Search and sequence analysis tools services from EMBL-EBI in 2022. *Nucleic Acids Res* 50, W276-W279. doi: 10.1093/nar/gkac240.

Scheich, C., Niesen, F. H., Seckler, R., and Büssow, K. (2004). An automated in vitro protein folding screen applied to a human dynactin subunit. *Protein Sci* 13, 370–380. doi: 10.1110/ps.03304604

Waterhouse, A. M., Procter, J. B., Martin, D. M. A., Clamp, M., and Barton, G. J. (2009). Jalview Version 2--a multiple sequence alignment editor and analysis workbench. *Bioinformatics* 25, 1189–1191. doi: 10.1093/bioinformatics/btp033
